# Heterogeneity in transmission parameters of hookworm infection within the baseline data from the TUMIKIA study in Kenya

**DOI:** 10.1186/s13071-019-3686-2

**Published:** 2019-09-16

**Authors:** James E. Truscott, Alison K. Ower, Marleen Werkman, Katherine Halliday, William E. Oswald, Paul M. Gichuki, Carlos Mcharo, Simon Brooker, Sammy M. Njenga, Charles Mwandariwo, Judd L. Walson, Rachel Pullan, Roy Anderson

**Affiliations:** 1 London Centre for Neglected Tropical Disease Research, Department of Infectious Disease Epidemiology, Imperial College London, St Mary’s Campus, London, W2 1PG UK; 2 MRC Centre for Global Infectious Disease Analysis, Imperial College London, St Mary’s Campus, London, W2 1PG UK; 3 The DeWorm3 Project, The Natural History Museum, London, SW7 5BD UK; 4 Faculty of Infectious and Tropical Diseases, London School of Hygiene & Tropical Medicine, London, WC1E 7HT UK; 5 Eastern & Southern Africa Centre of International Parasite Control (ESACIPAC), Kenya Medical Research Institute (KEMRI), Nairobi, Kenya; 6 Bill & Melinda Gates Foundation, Seattle, WA USA; 7 Departments of Global Health, Medicine (Infectious Disease), Pediatrics and Epidemiology, University of Washington, Seattle, USA

**Keywords:** Soil-transmitted helminths, Heterogeneity, Model fitting, Parasite aggregation

## Abstract

**Background:**

As many countries with endemic soil-transmitted helminth (STH) burdens achieve high coverage levels of mass drug administration (MDA) to treat school-aged and pre-school-aged children, understanding the detailed effects of MDA on the epidemiology of STH infections is desirable in formulating future policies for morbidity and/or transmission control. Prevalence and mean intensity of infection are characterized by heterogeneity across a region, leading to uncertainty in the impact of MDA strategies. In this paper, we analyze this heterogeneity in terms of factors that govern the transmission dynamics of the parasite in the host population.

**Results:**

Using data from the TUMIKIA study in Kenya (cluster STH prevalence range at baseline: 0–63%), we estimated these parameters and their variability across 120 population clusters in the study region, using a simple parasite transmission model and Gibbs-sampling Monte Carlo Markov chain techniques. We observed great heterogeneity in *R*_*0*_ values, with estimates ranging from 1.23 to 3.27, while *k-*values (which vary inversely with the degree of parasite aggregation within the human host population) range from 0.007 to 0.29 in a positive association with increasing prevalence. The main finding of this study is the increasing trend for greater parasite aggregation as prevalence declines to low levels, reflected in the low values of the negative binomial parameter *k* in clusters with low hookworm prevalence. Localized climatic and socioeconomic factors are investigated as potential drivers of these observed epidemiological patterns.

**Conclusions:**

Our results show that lower prevalence is associated with higher degrees of aggregation and hence prevalence alone is not a good indicator of transmission intensity. As a consequence, approaches to MDA and monitoring and evaluation of community infection status may need to be adapted as transmission elimination is aimed for by targeted treatment approaches.

## Background

Soil-transmitted helminths (STH) are the most prevalent of the neglected tropical diseases (NTD), infecting up to 1.5 billion people world-wide. The STH group comprises whipworm (*Trichuris trichiura*), roundworm (*Ascaris lumbricoides*) and hookworm (*Ancylostoma duodenale* and *Necator americanus*), but the majority of the global health burden results from hookworm species, which are estimated to account for the loss of approximately 5.2 million disability-adjusted life years [1]. The current WHO approach is to achieve control of STH infections through programmes of mass drug administration (MDA) targeted at school-aged and pre-school-aged children as well as other high-risk groups [2]. Recently, however, there has been an increased interest in the possibility of interrupting transmission through a short period of intensified community-wide MDA. Several recent and ongoing studies are currently testing this hypothesis [3, 4].

For soil-transmitted helminths, both prevalence and intensity are key epidemiological measures of community infection status for policy and programmatic decision-making. Within WHO guidelines, prevalence determines whether treatment is given, at what frequency and for how long [2, 5]. Severity of infection, as measured by the faecal egg count of infected individuals, is used as a proxy for worm load and infection-induced morbidity. The latter is a key element in cost effectiveness calculations [6–8]. However, within larger geographical regions, a great deal of heterogeneity is observed in the measured prevalence and intensity of STH infection. This may reflect variations in environmental conditions such as temperature and humidity, differences in social mixing or hygiene practices within the human host population, or the impacts of differing past MDA coverages [9, 10]. Spatial heterogeneity in prevalence and intensity makes it hard to predict how a region will respond to a control or elimination intervention based on MDA or WASH (water, sanitation and hygiene) improvements. As most countries enter a stage of high MDA coverage, at least in pre-school-aged and school-aged children, understanding the effects of MDA on STH infections is a prerequisite for the evaluation of the possible interruption of parasite transmission at the community level.

This study analyses the spatial variability in hookworm prevalence and intensity in a collection of contiguous communities taken from the baseline of a cluster-randomized trial conducted in coastal Kenya to evaluate treatment strategies for the soil transmitted helminths [3]. We seek to characterize the variability in the prevalence and intensity in terms of key epidemiological parameters, such as the basic reproduction number (*R*_*0*_) and the degree of parasite aggregation (as measured inversely by the negative binomial parameter *k*) in the human host population. The analysis is based on fitting a disease transmission mathematical model to the baseline data. This approach ensures that the resulting parameter values reflect the disease transmission processes found in endemic (or approximately endemic) parasite populations.

Our method puts constraints on possible parameter fits that are not present in purely statistical approaches to analyses [11–14]. Mathematical models of macroparasite infection predict ‘breakpoints’ in transmission created by the dioecious nature of helminths and the concomitant need for both male and female parasites to be in the same host to generate viable infective stages [15]. There exist prevalence and worm burden thresholds below which parasite populations cannot persist due to low mating success. Threshold values are strongly dependent on the degree of parasite aggregation and transmission intensity, as measured by *R*_*0*_ [16, 17].

Based on the models that describe parasite transmission, we can associate observed prevalence and intensity levels with the parameters that quantify the transmission cycle of the parasite and the diagnostic techniques used to measure the epidemiological quantities of prevalence and intensity of infection. By fitting the model to data on prevalence and intensity of infection to all clusters independently, we can examine the variability in parameter values to see how much can be explained by cluster-level environmental and demographic correlates. Importantly, a model fitted to data in this way can then be applied to directly investigating how prevalence and intensity in the region will evolve with time in individual clusters under different regimes of MDA treatment.

## Methods

### Epidemiological data

The TUMIKIA trial was initiated in 2015 with the aim of evaluating school *versus* community-based deworming on STH transmission in Kwale county, coastal Kenya [3, 18]. The study comprises three arms; namely, a control group of annual school-based de-worming, a group with annual community-wide deworming, and a third group with community-wide deworming biannually. The data used in this analysis comes from the baseline survey.

In the decade prior to the baseline survey of the study, this region received several rounds of lymphatic filariasis (LF) treatment (in 2003, 2005, 2008 and 2011), employing diethylcarbamazine citrate (DEC, 6 mg/kg) plus albendazole (400 mg), through the National Programme for Elimination of Lymphatic Filariasis (NPELF) [19]. Furthermore, from 2012 through 2014 annual school-based deworming with albendazole (400 mg) occurred through a programme, run by the Kenyan Ministries of Health and Education, to deworm all school-aged and pre-school-aged children living in high STH risk areas [20]. However, there is strong anecdotal evidence that the effective coverage levels for the prior rounds of treatment are significantly lower than those officially recorded (perhaps averaging 30% in reality).

The baseline survey was conducted in 120 contiguous study clusters, each comprising approximately 1000 households or 5000 individuals. We used the cross-sectional hookworm data from the study baseline, determined using duplicate Kato-Katz slide readings from a single stool sample. Sample sizes from clusters ranged from 110 to 294 individuals of all ages, selected at random from within randomly-selected households. The overall district prevalence of hookworm infection across the study site was 19% based on Kato-Katz diagnostics, with infection observed in 119 of the 120 study clusters. Prevalence at the cluster level ranged from 0% to 62.7%. Mean infection intensity across the district was 162 eggs per gram (epg), ranging from 0 to 726 epg. For the purposes of model fitting and cluster parameter estimation, we used hookworm prevalence and count data from each of the 119 clusters with non-zero prevalence.

**Fig. 1:**
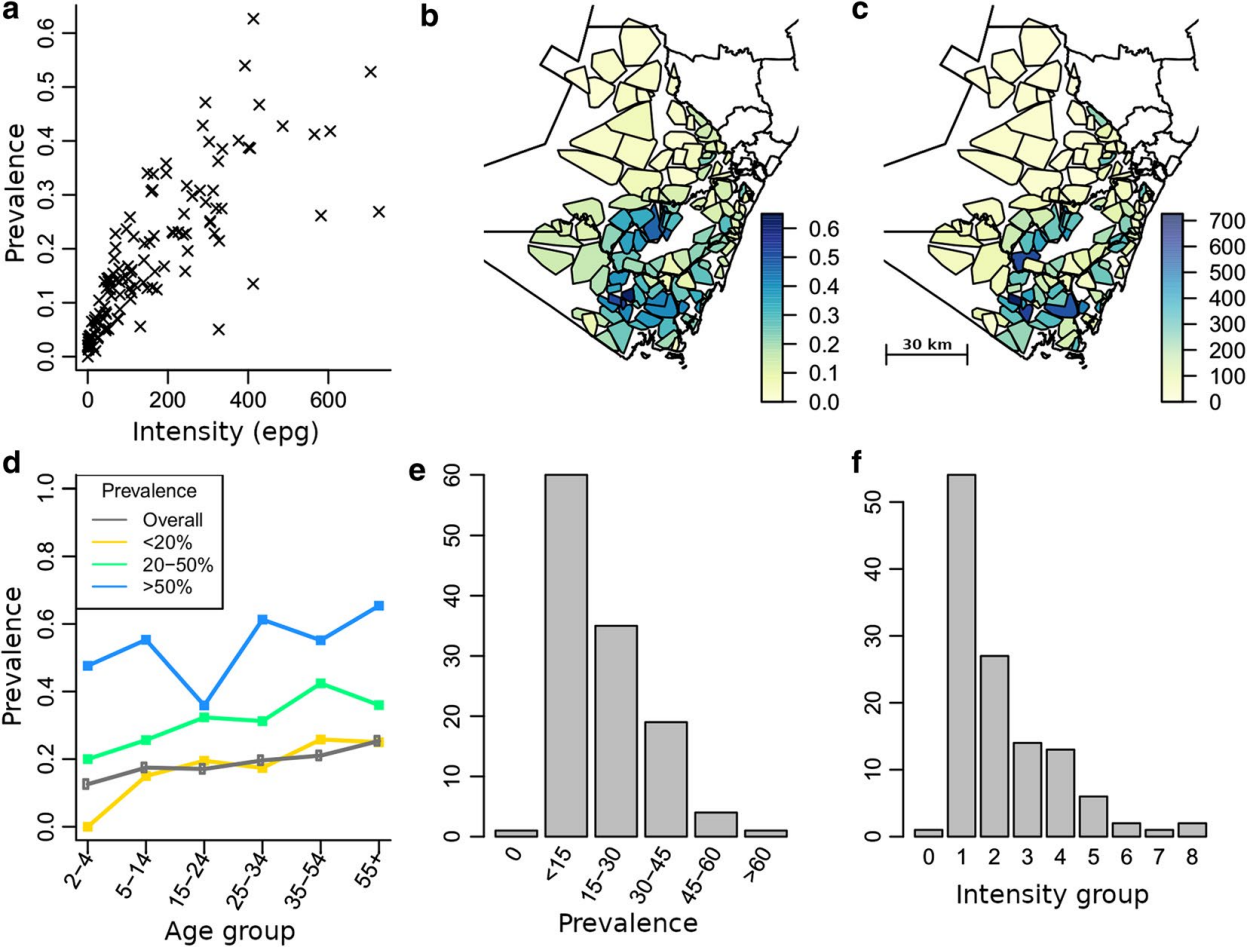
Summary statistics of hookworm epidemiological data from TUMIKIA study baseline, Kwale district. **a** Relationship between mean egg intensity and prevalence by cluster. **b** and **c** Regional maps of clusters in Kwale district with associated cluster level prevalence and mean egg intensity, respectively. **d** The typical age profiles of a cluster from each of the WHO prevalence ranges and the overall prevalence age profile of Kwale district. **e** and **f** Histograms of cluster prevalence and mean cluster intensity across all clusters, respectively. Egg intensity classes for panel **f**: 0, zero epg; 1, 0–100; 2, 100–200; 3, 200–300, etc

The prevalence/mean intensity data (Fig. 1c) show a correlation between egg count and measured prevalence in clusters. As might be expected, increasing prevalence is associated with a rising mean egg count in a cluster. There is evidence of the prevalence increase saturating to an upper bound as mean egg count increases to high values as predicted by the negative binomial model of the distribution of parasite numbers per host [21]. These observations are consistent with observations from a range of NTDs across large-scale heterogeneous populations [11, 12]. A few outliers exist with respect to the predicted negative binomial relationship between prevalence and intensity, arising in clusters that have anomalously large intensity measures for their measured prevalence. In the most obvious case, this is due to a single subject having an abnormally high intensity measure. Figure 1b, c shows the geographical distribution of clusters in Kwale district and their prevalence and mean infection intensities. The human population is concentrated in the southern and coastal areas of the district and in these more populous areas hookworm is typically the dominant STH infection.

Individual cluster age-stratified profiles show some evidence of an increasing infection intensity profile with age, as is typically observed and predicted by a simple infection and parasite mortality framework where the force of infection (FOI)—rate at which hosts acquire parasites per unit of time—is constant with age [22]. However, small sample sizes in individual age categories obscure this trend (Fig. 1d). Analysis showed that although there is evidence for a slight upward trend with age across the study site, for individual clusters there is no strong case for rejecting the assumption that the age profiles are flat and that infection status is independent of age (Additional file 1: Text S1, Figure S1a, b). This allows us to omit age structure from our deterministic model, which reduces the number of parameters required to describe the transmission dynamics. Figure 1e, f shows the distribution of prevalence and intensity across clusters, with both prevalence and intensity having distributions skewed towards low values. Mean and variance in prevalence fall within the range found in worldwide surveys of geographical variability in hookworm infection [23]. Infection intensity values are low to moderate, with only 0.5% of individuals with egg output categorized as heavy infection (≥ 4000 epg) [24].

### Mathematical model fitting method

The modelling approach uses two sources of data from the baseline survey of each cluster; the measured prevalence and the mean egg count. Although more extensive data are available from the TUMIKIA trial, prevalence and intensity data are adequate for fitting the transmission model to estimate key population parameters and to reflect the type and spatial scale of data that are generally available.

We analyzed the relationship between the measured prevalence and intensity in each cluster at baseline and the variation of these quantities across all clusters in the study. The current approach is an extension to simpler models in the literature which do not take into account the dynamic nature of parasite transmission [12–14, 25]. Our analysis is based on a simple parasite transmission model, coupled with a model of the diagnostic process which links faecal egg counts and prevalences to worm loads in the human host, taking account of density dependence in parasite fecundity.

Our dynamic parasite transmission model assumes that the parasite population in the hosts was in a stable endemic state, prior to the known history of MDA interventions preceding the baseline time-point. For hookworm, as for all other human helminth infections, there is a lower unstable equilibrium (a transmission breakpoint) below the stable endemic state which acts as a boundary between parasite persistence and extinction [15]. As noted earlier, transmission breakpoints are the result of the intra-host sexual reproduction of parasites, whereby low parasite prevalence in a host population make it unlikely for male–female pairs to found together in a host. The output of fertile eggs is thus reduced, further lowering the parasite population abundance and breaking the transmission cycle. A critical element in the position of the breakpoint is the degree of aggregation of worms among the host population as measured inversely by the negative binomial parameter *k*. As a result, the requirement for the parasite population to have been in a stable endemic state (prior to any pre-baseline treatment) places constraints on the possible sets of parameter values that the transmission model can have.

It is important to note that the recent history of chemotherapeutic treatment (i.e. past MDA coverage by age group) is an important determinant of the observed prevalence and intensity levels. In general, prior treatment will reduce prevalence and intensity levels at baseline below the stable endemic levels. If *R*_*0*_ is estimated assuming an endemic state, an artificially low value will be found. Recent epidemiological analyses have shown how to take into account the impact of prior treatment history on baseline estimates of *R*_*0*_ [26]. With the expansion of large-scale and national NGO treatment programmes, significant prior treatment is increasingly common. Indeed, it is an integral part of the DeWorm3 study (funded by the Bill and Melinda Gates Foundation), a large community cluster randomized trial being conducted in Benin, India and Malawi to determine the feasibility of interrupting STH transmission using school *versus* community-wide MDA [4]. Using a simple parasite transmission model that excludes age structure, given that the force of infection by age appears to be roughly constant for hookworm infections, allows us to include any known history of treatment and hence adjust for its effects in the estimation of parameters.

The model for the dynamics of worm burden excluding age structure in each cluster is given by

1 $$\frac{dM}{dt} = \sigma \left( {R_{0} f\left( {M;k,\gamma } \right) - 1} \right)M$$

as described in the works of Anderson & May [15, 17, 21].

In this equation, *1/σ* is the lifespan of the mature adult parasite in the human host and the parameter γ determines the severity of density dependence acting on egg production in female worms. The variable *M* is the mean female worm burden in the human host. Worms are assumed to be distributed among hosts according to a negative binomial distribution with aggregation parameter *k*. Given the lack of evidence for age structure in individual clusters in the TUMIKIA baseline data (Additional file 1: Text S1 and Figure S1b) and for the sake of model simplicity, the age dependency of worm burden has been suppressed given that (as noted earlier) observed age intensity of infection profiles suggest a fairly constant force of infection across age classes. The function *f* is given by

$$f\left( {M;k,z} \right) = \frac{1}{{\left( {{{1 + M\left( {1 - z} \right)} \mathord{\left/ {\vphantom {{1 + M\left( {1 - z} \right)} k}} \right. \kern-0pt} k}} \right)^{k + 1} }}\left[ {1 - \left( {\frac{{{{1 + M\left( {1 - z} \right)} \mathord{\left/ {\vphantom {{1 + M\left( {1 - z} \right)} k}} \right. \kern-0pt} k}}}{{{{1 + M\left( {2 - z} \right)} \mathord{\left/ {\vphantom {{1 + M\left( {2 - z} \right)} k}} \right. \kern-0pt} k}}}} \right)^{k + 1} } \right]$$

where $z = \exp \left( { - \gamma } \right)$ [16]. The first term on the right-hand side represents the mechanism of density dependence which limits egg output due to overcrowding of parasites within the host. The second term represents the impact of parasite sexual reproduction within the host assuming hookworms are dioecious and polygamous, reducing the output of fertilized eggs due to scarcity of a mate at low mean burdens of infection.

The mean worm burden dynamics of this model is linked to measured prevalence and intensity based on faecal egg counts through a model for egg count diagnostics and the relationship with worm load. The mean egg count as a function of fertilized female worms in the host is given by $\bar{E} = \lambda n_{f} \exp \left( { - \gamma n_{f} } \right)$, where λ is the net egg output for a fertilized female and γ parameterizes the drop in fecundity with increasing worm burden. As is well known in population ecology, the density-dependent fecundity mechanism limits the reproduction of the worms, leading to the existence of a stable endemic population. Measured egg counts are negative-binomially distributed with mean $\bar{E}$ and aggregation parameter *k*_*e*_ (*k*_*e*_ is not the same as the worm aggregation parameter, *k*) [27, 28]. For a given mean worm burden, this distribution allows us to estimate the probability distributions for measured prevalence and the total faecal egg intensity in a population. This enables us to construct a likelihood for the TUMIKIA baseline data. If the baseline data is described by pairs of data {*P*_*i*_, *E*_*i*_} for the i^th^ cluster, the total likelihood for the data is

$$L_{T} = \prod\limits_{i}^{N} {\pi \left( {P_{i} ;M_{i} ,\theta_{i} } \right)} I\left( {E_{i} ;M_{i} ,\theta_{i} } \right)$$

where *π*(*P*_*i*_*;M*_*i*_*,*_*i*_) and *I*(*E*_*i*_*;M*_*i*_*,*_*i*_) are the probabilities of measured prevalence *P*_*i*_ and total intensity *E*_*i*_, given a model mean worm burden, *M*_*i*_ and parameters *θ*_*i*_. The details of the model and likelihood calculations are provided in Additional file 2: Text S2.

In the parameterization of the model, it is important to distinguish between mechanisms that are common to all clusters and those that may vary amongst them. Global parameters across all clusters include diagnostic parameters [the mean measurable egg output from a single fertilized female worm, *λ*, the aggregation parameter for egg output, *k*_*e*_, parasite life-cycle parameters (the density-dependent fecundity parameter *γ* and the mean lifespan of hookworm, 1/σ]. Each cluster has specific values for *R*_*0*_ and worm aggregation, *k*. These parameters vary across clusters. Values of *k* are constrained to be proportional to the measured prevalence of a cluster in line with previous observations [12, 13]. The aggregation in the i^th^ cluster is defined as

$$k_{i} = k\left( {P_{i} } \right) = k_{L} + \frac{{\left( {P_{i} - 0.1} \right)}}{0.5}\left( {k_{U} - k_{L} } \right)$$

where *P*_*i*_ is the measured prevalence in the *i*^*th*^ cluster and *k*_*L*_ and *k*_*U*_ are the values of *k* at prevalences of 10% and 60%, respectively.

Due to the large number of *R*_*0*_ parameter estimates to be derived for each cluster in the fitting process, we employ a Gibbs sampling approach to investigating the likelihood distribution. Since the *R*_*0*_ contributions to the likelihood are largely independent of each other, this allows a faster and a more stable investigation of the likelihood distribution.

### Assessment of covariates contributing to *R*_*0*_ heterogeneity

Open source data for mean annual temperature and annual rainfall [29], elevation [30], population density [31], and land cover [32] were used in the analysis of possible associations with the prevalence of hookworm infection [29, 30]. Cluster level access to sanitation and principal components analysis-derived wealth scores were calculated from the TUMIKIA dataset by taking the percentage of households reporting access and mean PCA wealth category, respectively. Cluster boundaries were formed through the convex hull of all household GPS locations. Cluster level data scores were determined by averaging all pixels within a cluster boundary for temperature, rainfall, elevation, and population density. For each cluster, the percentage of each classification of land cover was used. The 16 land cover classifications used include the following: cropland, irrigated cropland, herbaceous cover, mosaic cropland/natural vegetation, mosaic natural vegetation/cropland, tree cover (evergreen), tree cover (mixed leaf), tree cover (deciduous), flooded tree cover (freshwater), flooded tree cover (saline water), herbaceous cover/tree or shrub, mosaic tree and shrub/herbaceous cover, shrub land, grassland, water bodies, and urban area.

To quantify what each indicator could account for in the observed variance in *R*_*0*_ estimates, both alone and in combination, GLM models were employed using scaled cluster-level covariate values. To assess every combination of the 22 covariates, we ran all model combinations but limited the number of covariates included in each model from 1 to 8. The goodness of fit was assessed *via* AIC and pseudo-*R*^2^ using the *caret* package in R [33].

## Results

We first present results for the maximum likelihood estimator (MLE) parameter values with a fixed value of the parameter γ, which controls the density dependence of egg production. This serves to illustrate the main qualitative features of the fitted parameters and their relationship to the observed epidemiological data.

**Fig. 2:**
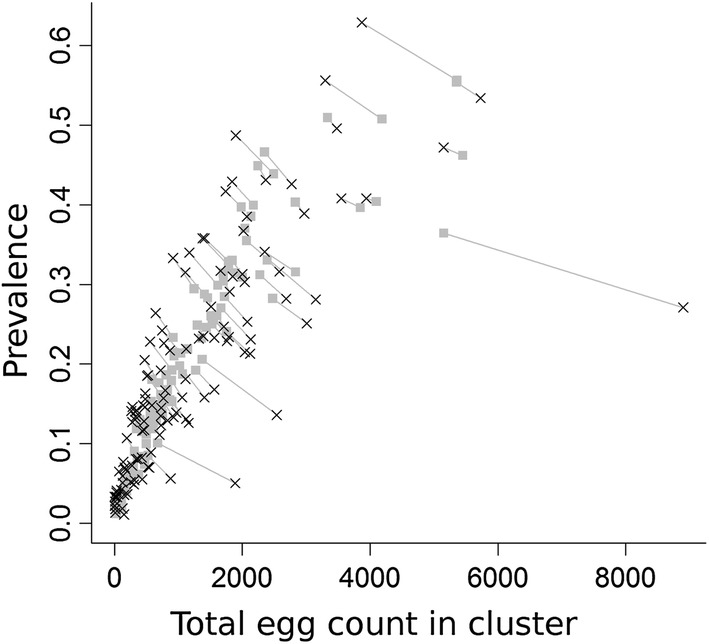
Observed prevalence and total egg count for each cluster against mean model output for MLE parameter values. Grey squares represent model results and black crosses represent the data. Points from the same cluster are joined by a grey line

Figure 2 shows prevalence and egg count data and the matching model predictions for each cluster for the MLE parameter set. The data and prediction for each cluster are joined by a line. We observe a good concordance between measured prevalence and egg count data and our model predictions by cluster. The model fit captures the trend/relationship defined by the negative binomial probability model between prevalence and egg count as well as the saturation in prevalence for higher egg counts. Allowing *R*_*0*_, and *k* to vary between clusters means that the model can capture much of the data variability around the main trend line.

**Fig. 3:**
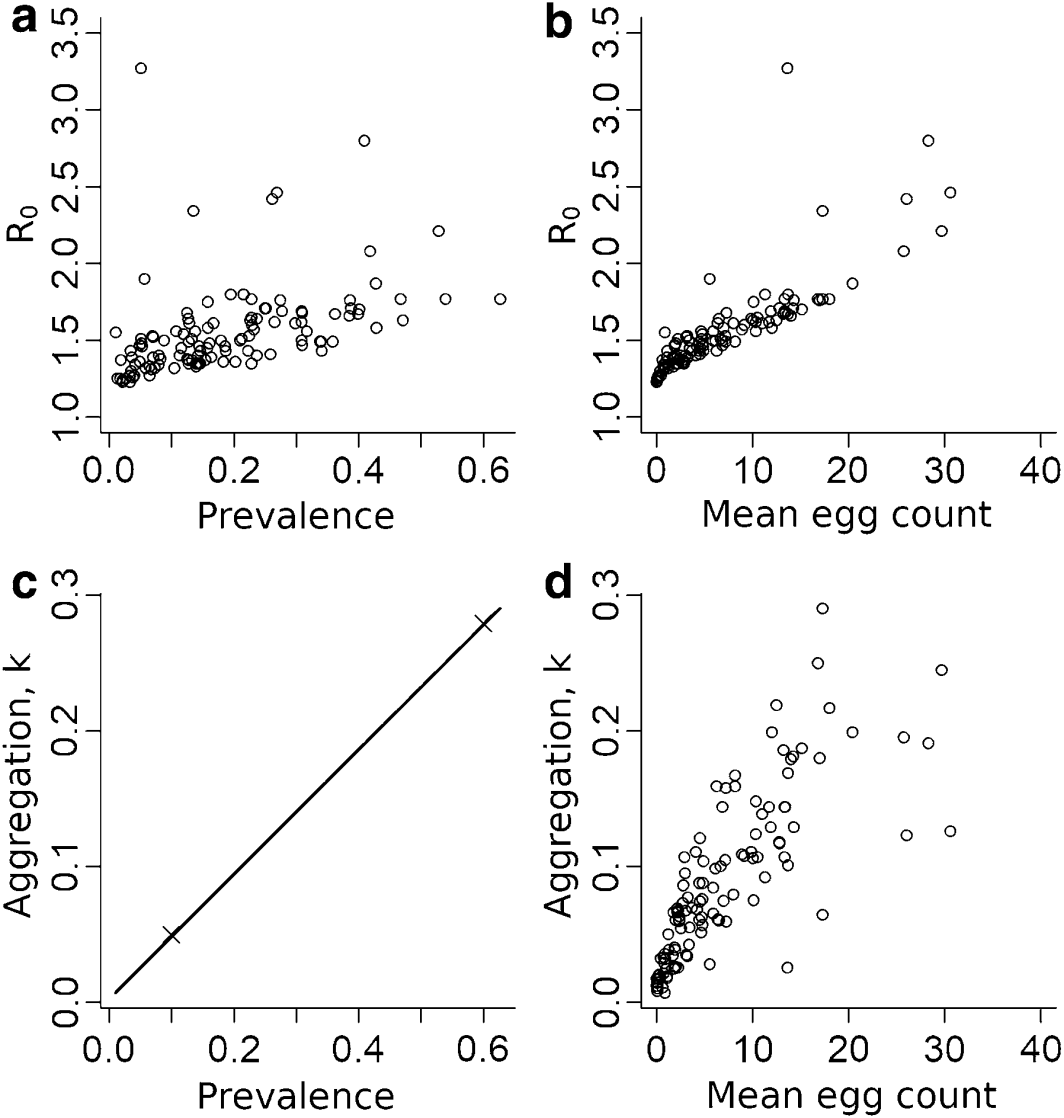
Cluster-level relationship between *R*_*0*_ and *k* for the model and prevalence and mean egg count. **a** Cluster-level MLE *R*_*0*_ *versus* measured prevalence. **b** Cluster-level MLE *R*_*0*_ *versus* observed mean egg count by cluster. **c** Aggregation, *k*, *versus* measured prevalence. **d** Aggregation, *k*, *versus* observed mean egg count by cluster

The best-fit for cluster aggregation parameters (*k*) shows a strong dependence on the measured prevalence of the cluster (Fig. 3c). The recorded pattern is a result of the constraint on *k*-values described in the Methods section, with the crosses in the figure showing the position of the two parameters defining the line at prevalence values of 10% and 60%. In low prevalence clusters (P < 10%), *k*-values of 0.05 or lower are observed. The lowest found in using the MLE parameters is approximately 0.007, corresponding to a measured infection prevalence of about 1%. Since the aggregation parameter *k* is positively correlated with prevalence at the transmission breakpoint, it is possible that the low prevalence *k*-values are principally driven by the need within the parameter estimation process to achieve a stable endemic disease state at very low prevalence. For the highest prevalence clusters (around 60% measured prevalence), *k*-values rise to around 0.3. This value is in line with previous analyses of hookworm aggregation in untreated communities [17]. Worm expulsion studies report results in the approximate range of 0.25–0.60 [34–36]. Such studies have been typically carried out in communities with high prevalence and little or no past drug treatment.

Cluster-specific *R*_*0*_ MLEs show only a weak correlation with measured prevalence (Fig. 3a); values range from 1.23 to 3.3. There is a general upward trend in *R*_*0*_ for increasing measured prevalence, but a wide range of *R*_*0*_ estimates can be found for any given narrow range of prevalence values. This variability in *R*_*0*_ is not the result of the model failing to fit to the prevalence data, as shown in Fig. 2. As will be discussed later, prevalence within this type of model is not strongly sensitive to estimates of *R*_*0*_. As shown in Fig. 3b, the correlation between measured intensity and *R*_*0*_ is higher, indicating a greater sensitivity of mean intensity to the value of the reproductive number. This is to be expected based on past work on models of the transmission dynamics of STH species, where *R*_*0*_ is predicted to be linearly related to mean worm load and prevalence saturates quickly as *R*_*0*_ rises. The relationship between aggregation, as measured by *k*, and mean intensity shows only a very weak correlation (Fig. 3d).

**Fig. 4:**
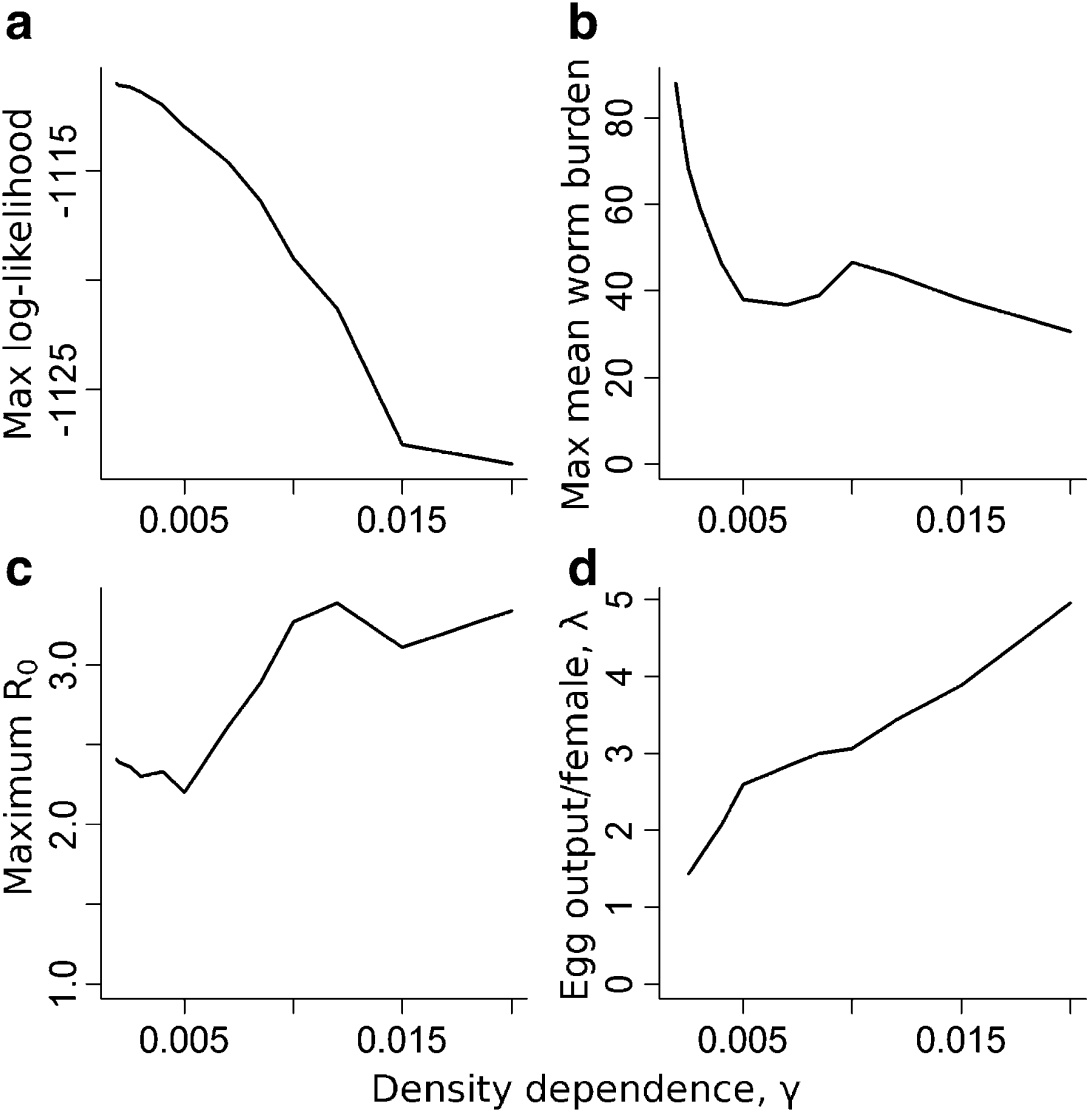
Sensitivity of the estimates of key parameters and other aspects of the model to the value of gamma, which measures impact of individual worm burden on worm fecundity. **a** Maximum likelihood. **b** Maximum mean total worm burden across all clusters. **c** Maximum *R*_*0*_ value across all clusters. **d** Egg output per female worm in the absence of density dependence, λ

The results discussed so far are based on a fixed value for the density-dependent fecundity parameter, γ = 0.01. If γ is allowed to vary freely, a best-fit value of around 0.002 is derived. However, a difference of only 15 separates the maximum likelihood at this value from that at 0.02 (see Fig. 4a). Spread across the 119 clusters, the loss in quality of fit is negligible. The dependence of aggregation on prevalence remains fairly robust across different values of γ. However, the model also predicts that the mean female worm burden in a cluster to be strongly dependent on γ, with the maximum mean worm burden of over 80 for γ = 0.002. This value increases rapidly for low values of γ as shown in Fig. 4b. A brief survey of mean hookworm burdens from worm expulsion studies can be found in Turner et al. [37]. Worm burdens are broadly uniform with age except for the very young children, suggesting a constant age-independent FOI, with the highest burdens being around 40–50 worms per person. If the accuracy of the expulsion counts is reasonably good, the data suggest that worm burdens above 40–50 are very rare and hence γ values should be bounded below that value of about 0.005. The *R*_*0*_ values across all clusters broadly increase with increasing γ (Fig. 4c), reflecting the decreasing net production of fertile eggs as the severity of density dependent fecundity rises. This effect is partially offset by the increasing net fecundity of female worms with increasing γ, allowing fewer worms to generate a given output of fertile eggs (Fig. 4d).

Information on the possible ranges of realistic parameter values can be inferred from the shape of the likelihood distribution. The properties and interpretation of the likelihood distribution are discussed in detail in the Additional file 3: Text S3. The likelihood distribution has an unusual shape as a consequence of the nature of the model (see Additional file 3: Figure S2). First, the distribution is quite skewed, in that the MLE parameters generally fall at the outer limit of credible intervals defined from the likelihood sample. This feature is a consequence of a major non-linearity within the model structure, namely, the breakpoint in transmission created by the sexual mating function and the requirement that the disease state prior to treatment should be a stable endemic state. Endemic states in areas of low transmission can be close to parasite-free states of the model (i.e. the second stable equilibria, separated from the stable state of endemic infection by the unstable breakpoint in transmission) and these have very low likelihoods. For example, the best-fit solutions often have low *R*_*0*_ values, but this places them close to parameter sets at which endemic solutions do not exist. As a result, in exploring the parameter space of likelihoods, the majority of ‘time’ is spent at higher *R*_*0*_ values which are not close to critical values, although they have lower likelihoods. The same effect can be seen in the estimation of the *λ* and the *k* parameters which are highly correlated with the value of *R*_*0*_.

A second feature is that most parameter values sampled by the Monte Carlo Markov chain (MCMC) chain are much lower than the maximum likelihood. The distribution of log-likelihood values is approximately ^2^ in distribution with degrees of freedom equal to the number of parameters fitted. With more than 120 parameters, the most frequently appearing log-likelihood values in the likelihood sample are far below the maximum value, by a difference of approximately 100 (Additional file 3: Figures S2 and S8).

**Fig. 5:**
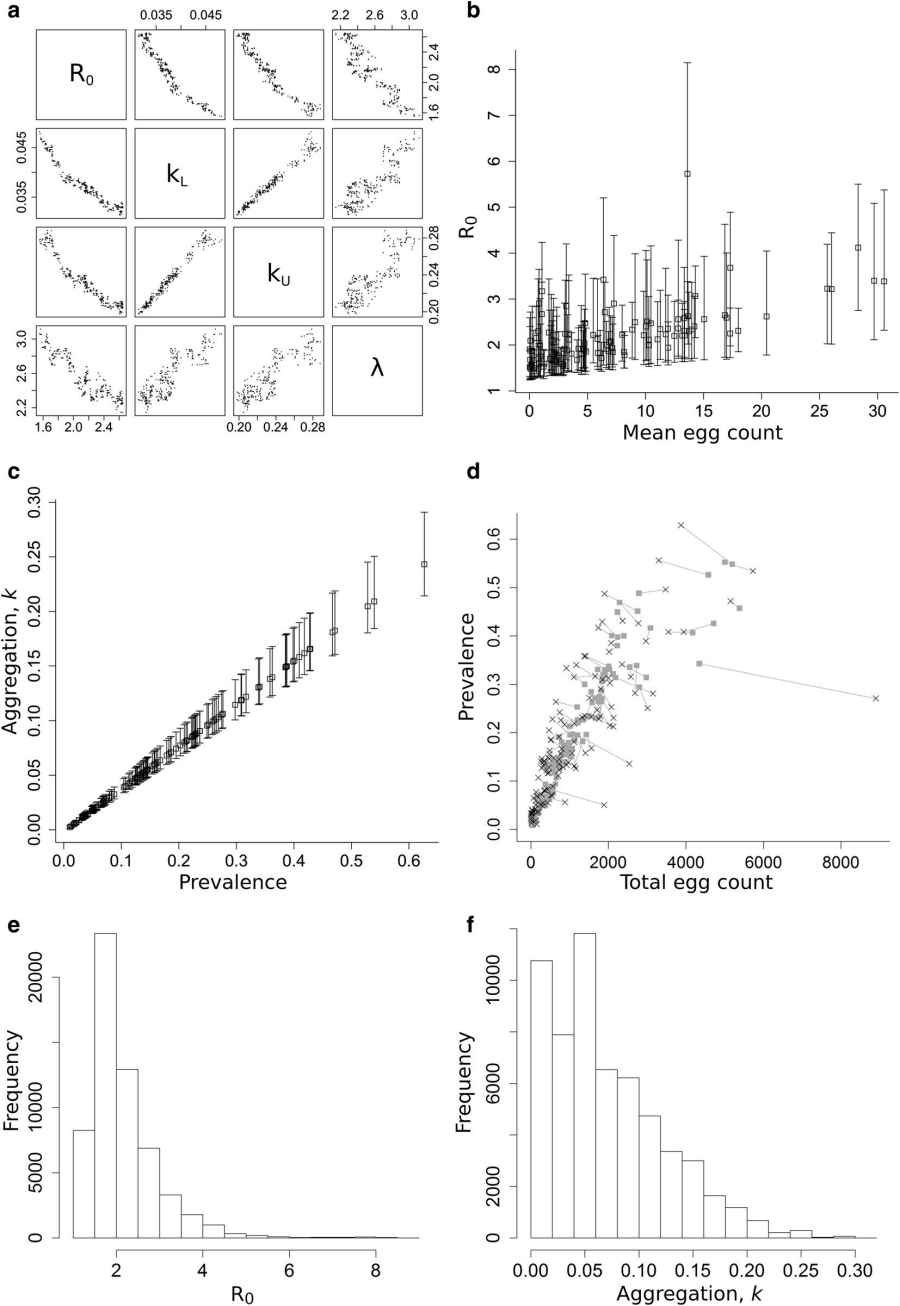
Parameter distribution and fit to data for the likelihood distribution truncated below *−* 1190. **a** Range and correlations of fitted parameters. **b** Mean and 95% CIs for *R*_*0*_ values against mean egg count of clusters. **c** Mean and 95% CIs for aggregation parameter *k* against measured cluster prevalence. **d** Model fit to data for parameter set constructed from mean values taken from the truncated likelihood. Log-likelihood = − 1156. **e** and **f** Distribution of *R*_*0*_ and *k*, respectively, across the truncated likelihood

Despite the unusual structure of the likelihood distribution, the fit to data it represents is generally good across the majority of clusters. However, for a minority of clusters the observed prevalence and intensity data lies outside the range predicted by the model (see Additional file 3: Figures S4 and S5). A particular problem with the model over a large range of parameter sets sampled from the likelihood distribution is the large predicted mean worm burden, analogous to the problem noted in the previous section with respect to sensitivity to the fecundity parameter, *γ*. Mean worm burden in the model is effectively a ‘hidden variable’ in that it is not directly measured, and no data is directly associated with it. As a result, parameter sets that give very different mean worm burdens can result in very similar likelihood values for the data. Taking the average parameter values from the MCMC sample as a parameter set, the resultant maximum mean worm burden among clusters is around 350, with a log-likelihood for the data of − 1208, which is about 90 units below the maximum. High worm burdens are generated by large values of *R*_*0*_ in the parameter set (several clusters have *R*_*0*_ > 15; see Additional file 3: Figure S6). A simple way to exclude parameter sets that give rise to large worm burdens is to truncate the likelihood distribution at a minimum log-likelihood value. The strong positive correlation between log-likelihood and *R*_*0*_ then limits the maximum worm burdens in the remaining distribution. A minimum LL value of − 1190 restricts maximum mean worm burdens to below about 80 per host. Figure 5 shows the distribution of parameter values within the truncated likelihood distribution and a representation of the fit to data for the mean parameter values from the truncated distribution.

Figure 5a shows that there is a strong linear correlation between the fitted parameters in the truncated likelihood. This correlation starts to break down for log-likelihood values lower than the cut-off point. *R*_*0*_ values show a great deal of variability within individual clusters (Fig 5b), but there is a clear increasing linear trend in mean *R*_*0*_ values with mean egg count as predicted by simple models of STH transmission dynamics [17]. The corresponding trend in *R*_*0*_ values *versus* cluster prevalence is much less clear. Again, simple models suggest prevalence saturates at a level determined by the aggregation parameter *k* (low prevalence for low *k*-values) and as such a strong association is not expected.

The variability in the estimated values of *R*_*0*_ is a consequence of the high variance in the negative binomial egg production distribution. Overall, the goodness-of-fit of the model for parameter values taken from the truncated likelihood is quite good, as can be seen by comparing Fig. 5c, from the mean parameters from the truncated likelihood, with the equivalent MLE fit in Fig. 2. The log-likelihood difference between these two fits is only 37, which is small when distributed across the 119 clusters. Figure 5e, f shows the distribution of *R*_*0*_ and *k* across all clusters in the truncated likelihood approach. While high *R*_*0*_ values (*R*_*0*_ > 5) occur, 95% of values fall below 3.7. The information in Fig. 5 is summarized in Table 1. However, the table does not indicate the strong correlation between parameter ranges depicted in Fig. 5.

**Table 1:** Fitted (*λ*, *k*_*10*_, *k*_*60*_) and unfitted (*σ*, *ϒ*, *k*_*e*_*)* model parameters. MLE parameter values and ranges are shown for fitted parameters. *R*_*0*_ values are cluster specific and therefore omitted

Parameter	Definition	Fitted	MLE value	Range
*λ*	No. of eggs per fertilized female worm	Yes	3.06	2.2–3.06
*k*_*L*_	Aggregation at 10% prevalence	Yes	0.048	0.03–0.048
*k*_*U*_	Aggregation at 60% prevalence	Yes	0.278	0.14–0.278
*σ*	Adult worm life expectancy	No	2 years	–
*ϒ*	Density dependence fecundity	No	0.01	–
*k*_*e*_	Aggregation of egg output	No	0.8	–

When we compared the model-inferred *R*_*0*_ values with socioeconomic and climatic correlates, we found that *R*_*0*_ is negatively correlated with cluster level wealth scores and elevation, and positively correlated with mean annual precipitation and temperature [38–41]. Both results make intuitive sense for hookworm. When assessed alone, of the 22 climatic (precipitation, temperature, elevation), land cover (e.g. tree-cover, water bodies, cropland), and socioeconomic (mean PCA wealth, % access to sanitation, population density) indicators, mean annual precipitation can account for the most variation in *R*_*0*_ (*R*^2^ = 0.165). However, when statistical models are expanded to include up to 8 covariates, the *R*^2^ value can be increased to 0.32 through a combination of land cover and socioeconomic variables, see Table 2.

**Table 2:** Output for the best performing/fitting model, as assessed by AIC. Model combinations include up to 8 covariates from all 22 possible covariates. Additional file 4 documents a full list of covariates

	Covariate	Coefficient value (95% CI)
	Intercept	1.847 (1.551–2.142)
Socioeconomic	Mean wealth score	− 0.695 (− 1.071–− 0.319)
	Sanitation access	0.307 (0.155–0.46)
Landcover	Mixed leaf treecover	0.096 (0.02–0.172)
	Broad leaf evergreen tree cover	0.091 (0.035–0.148)
	Flooded saline-water tree cover	− 0.342 (− 0.603–− 0.081)
	Flooded freshwater tree cover	0.061 (0.01–0.112)
	Waterbodies	0.336 (0.076–0.597)
	Broadleaf deciduous tree cover	− 0.063 (− 0.114–− 0.012)

## Discussion

The TUMIKIA trial in Kenya has generated detailed epidemiological data for hookworm infection across a large geographical area. The high quality of the surveying and monitoring processes means that heterogeneity with regard to data measurement quality is minimized, allowing the heterogeneity arising from epidemiological processes to be studied more precisely. The dataset records a wide range of baseline hookworm prevalence values across clusters, spanning the low, medium and high categories as defined in WHO control guidelines for mass drug administration [5]. As commonly noted in large scale STH epidemiological studies, infection is often very focal in nature for reasons that are typically poorly understood. The prevalence range is comparable to that found in other surveys of heterogeneity within-country hookworm prevalence, although the distribution of recorded prevalence values in TUMIKIA is more skewed towards low prevalence given the past successes in getting good MDA coverage for both LF and STH control [23]. This skewedness may reflect the impact of past MDA programmes within certain cluster settings and/or adverse dry climatic conditions that are not conducive to larval hookworm survival in the external habitat. The county in which the TUMIKIA trial was conducted has variable climatic conditions and there is also variability in the community wealth and social structures of the population. Furthermore, given that clusters were formed from pre-existing community health units (the lowest level of health service provision in Kenya), they are not uniform in terms of spatial scale or population size. They differ in the number of villages which make up a cluster, the geographical scale (i.e. cluster area), and environmental factors (e.g. rural communities *versus* peri-urban). For example, in peri-urban areas cluster size can be as small as 2 km^2^, while in rural areas clusters can be upwards of 175 km^2^. The heterogeneity in transmission potential, *R*_*0*_, in a defined cluster setting may reflect some or all these factors. It is important to note that prevalence is related to the magnitude of *R*_*0*_, but in a non-linear manner due to the limiting effect of density-dependent fecundity. More importantly, prevalence is directly dependent on the degree of worm aggregation in the population, with increasing aggregation reducing the number of infected individuals.

A number of published studies have attempted to analyze prevalence-intensity data in order to understand worm aggregation and transmission intensity across a range of human helminth infections including STH and schistosomes [12–14, 21, 25]. Our analyses extend past approaches in several ways. By basing the analyses around a parasite transmission model, we can take prior rounds of MDA treatment into account. Previous chemotherapeutic interventions reduce prevalence and intensity of infection in communities which will lead to an underestimate of the basic reproductive number *R*_*0*_ at baseline. For example, recent research has shown that, for hookworm, LF MDA programs can greatly lower baseline hookworm prevalence [26]. For the TUMIKIA study site, however, prior LF treatment was likely too infrequent and limited to have much effect on hookworm prevalence and intensity prior to the collection of the baseline data in each cluster. By explicitly including a model of Kato-Katz diagnostic sensitivity [42], we are able to give an appropriate likelihood to observed epg or parasite count data (see Additional file 2: Text S2).

Model results for MLE parameters show a clear linear trend in *R*_*0*_ values increasing as mean egg count rises as predicted by simple theory. The estimated relationship with prevalence was non-linear as again predicted by simple theory (Fig. 3a and b, respectively) [17]. However, the variability in *R*_*0*_ values (Fig. 5b) is of the same order as the range of the MLE values across all clusters. The uncertainty is a consequence of the high variance of the probability distribution for individual egg counts. The model of Kato-Katz egg counts compounds a negative binomial distribution for egg output from a single host, compounded with the negative binomial distribution of worms amongst hosts. The predicted egg counts therefore have very high variance-to-mean ratios, as do observed egg counts for STH across populations. The high degree of uncertainty in modelled egg counts is reflected in sometimes-large disparities between the generated model mean egg counts and the measured data, which can be seen clearly in the model-data comparisons shown in Figs. 2 and 5d. Most of the discrepancy between MLE model predictions and data is found in the egg counts. Assuming no variability in model egg count, as is the case in previous models, would clearly have exerted a strong bias on the model fit and the resulting parameter ranges that are determined.

The multi-cluster model adopted allows some parameters to vary between clusters (i.e. *R*_*0*_ and *k*) while others remain fixed, on the assumption that they are fixed features of the parasite’s biology (i.e. fecundity, life expectancy and the severity of density dependence on fecundity). The clearest pattern arising from this type of analysis is the relationship between prevalence and the degree of parasite aggregation as measured inversely by the negative binomial *k*. As infection prevalence falls, the degree of worm aggregation among hosts increases. The relationship arises in part from the requirement for a stable endemic parasite population over a wide range of baseline prevalence values. Only a high degree of aggregation allows sexual reproduction to continue when the parasite population is low; without aggregation, males and females cannot meet to mate. However, very aggregated parasites make high prevalence hard to achieve without very large *R*_*0*_ values. Under these conditions, the model generates unrealistically large worm burdens in some clusters, leading to the rejection of those solutions. The negative correlation between prevalence and worm aggregation allows the model to encompass both high and low endemic prevalence values.

The present results suggest that as the FOI declines, heterogeneity in disease transmission increases between hosts. This effect could arise from many processes or a combination of them. One example is if there were multiple reservoirs of infectious material in the environment instead of just one. For example, if each household were to have its own infectious reservoir in addition to a background global reservoir, a drop in FOI could reduce the background FOI, leaving households to a greater extent re-infecting themselves and resulting in the aggregation of parasites within households. There is some evidence that within-household reinfection is a major contributor to parasite burden [43]. A test of these ideas could come from mid- or end-point data from the TUMIKIA trial, which would show the effect of multiple rounds of MDA on the same populations with unchanged social and environmental conditions. If aggregation is unchanged in clusters over time, this would suggest that FOI is not a driver of aggregation change, but that social and environmental conditions are. In any event, increased aggregation at low prevalence has clear implications for monitoring and evaluation of control surveillance after elimination. If aggregation reflects household structure, for example, it may be possible to identify key ‘sentinel’ households as indicators of parasite prevalence in the community. As mentioned earlier, another explanation lies in persistent non-compliance to treatment in a small fraction of people that results in reservoirs of infection.

Our analyses attempted to account for variation in *R*_*0*_ using cluster level climatic, socioeconomic and land cover data, with the aim of determining any covariates that may contribute to heterogeneity in disease suitability across clusters. The relatively small amount of *R*_*0*_ variation that mean annual temperature and elevation account for, and their absence from the best performing models, may be due to the narrow range of values across the study zone (range of 24.1–26.4 °C and 7–393 m, respectively). Indeed, the ranges of both mean annual temperature and elevation fall well within those suitable for hookworm larval viability [38, 44]. Interestingly, the opposing forces of wealth and access to sanitation on *R*_*0*_ suggest that the quality of the latrine that households have access to within the study area may increase transmission, rather than mitigating it [45]. The best-fitting models include multiple tree-cover variables, indicating that relative shade and drainage of soil may influence disease transmission, and consequently *R*_*0*_. The presence of water bodies or flooded areas within a cluster is positively correlated with *R*_*0*_. We are unable to account for sizable portion of the variation in *R*_*0*_, which may be due to the relatively small geographical area of the study site, one district of Kenya, and the narrower range in data values for each possible indicator compared to country-level values. Moreover, this may be a consequence of the inherent uncertainty of the MLE *R*_*0*_ estimation procedures adopted. The correlation between classical climatic covariates (e.g. temperature, precipitation, elevation) and *R*_*0*_, is uniformly less significant if performed with prevalence as opposed to *R*_*0*_ alone. This is to be expected as prevalence tends to plateau (mediated by the value of *k*) as *R*_*0*_ increases. What is key however, is that local climate is of importance in determining the success of hookworm transmission and hence could be of use in focusing MDA coverage of areas in which conditions are highly suitable for infection.

Our study shows that the spatial heterogeneity in prevalence and intensity is indicative of a matching heterogeneity in the epidemiological dynamics of the parasite within the human host population. Such heterogeneity has important consequences for policy formulation for morbidity and infection control, as well as programmes that aim at transmission elimination. These are normally implemented on spatial scales larger than that of the heterogeneity observed in the TUMIKIA study.

A natural approach is to design interventions to be effective against the highest transmission intensity ‘hot or focal spots’ or lowest compliers to treatment in an implementation unit, on the assumption that this will be efficacious against all locations in a region. However, as exemplified by the WHO guidelines for control of STH and other NTDs, intervention strategies are based on infection prevalence levels across an implementation unit. Variation in prevalence and mean intensity of infection within an implementation unit and the consequent variation in disease dynamics highlighted in this paper will lead to a range of responses to MDA. As a result, strategies aimed at the mean prevalence will likely fail in a significant number of areas within the implementation unit in terms of the frequency and coverage level of MDA required to either eliminate morbidity of interrupt transmission.

## Conclusions

The work presented in this paper shows that the link between prevalence and transmission intensity (*R*_*0*_) is not fixed but is critically dependent on the degree of parasite aggregation in communities. For a given value of infection prevalence in a population, parasite transmission intensity could vary considerably depending on the level of parasite aggregation within the human host population. Hence, prevalence alone may not be a reliable indicator of transmission intensity. This again has important policy implications for WHO in any revision of the STH control guidelines for the 2030 Roadmap targets. The high degree of parasite aggregation associated with low prevalence values after multiple rounds of MDA suggest that in the ‘end game’ of STH control once prevalence is low, different approaches to MDA distribution may be desirable. High levels of aggregation suggest that infection may be localized in small hotspots, possibly at the household level, or in groups who are consistently non-compliant to control. As such, novel approaches to identifying, monitoring and treating such hotspots and or non-compliers in order to maintain low prevalence or achieve a break in transmission, are required to avoid unnecessary treatment of a largely uninfected population.

## Supplementary information

**Additional file 1: Text S1.** An analysis of cluster level age profiles. **Figure S1. a** Total log-likelihood for prevalence gradient, m across all clusters (MLE: m = 0:0012/year). **b** Histogram of MLE prevalence gradients for the 119 clusters. Individual MLE values are subject to an uncertainty of *c.*0:0015/year.

**Additional file 2: Text S2.** Analysis and derivation of likelihood functions that connect the model to data.

**Additional file 3: Text S3.** Details of the likelihood distribution and the quality of fit of the model. **Table S1.** MLE and ranges for global parameters from truncated likelihood distribution. **Figure S2**. Marginal distribution of the likelihood sample. **Figure S3.** Joint parameter distribution for two individual clusters. **Figure S4.** Model mean prevalence and total egg count for each cluster. **Figure S5.** Distribution of model mean prevalences and total egg counts arising from likelihood sample for two individual clusters. **Figure S6.** Mean and 90% credible intervals for *R*_*0*_ and *k* for each cluster as sampled from the likelihood distribution. **Figure S7.** Comparison of model fit to data with MLE and mean parameters. **Figure S8.** Pairs plot of mean *R*_*0*_ and other parameters with and without likelihood cut-off. **Figure S9.** Mean and 90% credible intervals for parameter estimates with and without likelihood truncation. **Figure S10.** Correlation between *R*_*0*_ and *k* within the truncated likelihood sample. **Figure S11.** Fit to data for mean and MLE parameters from the truncated likelihood.

**Additional file 4: Text S4.** Full description of covariates for GLM model fitting. **Table S2.** Landcover (16 variables).

## Data Availability

Data analyzed in this study will be made available to members of the scientific and medical community for non-commercial use only, upon email request to RP. Data are stored in Data Compass, the London School of Hygiene & Tropical Medicine digital data repository, https://datacompass.lshtm.ac.uk/.

## Abbreviations

STH: soil-transmitted helminths
MDA: mass drug administration
NTD: neglected tropical disease
WASH: water, sanitation and hygiene
LF: lymphatic filariasis
NPELF: National Programme for the Elimination of Lymphatic Filariasis
epg: eggs per gram
FOI: force of infection
MLE: Maximum Likelihood Estimator
MCMC: Monte Carlo Markov chain
