# Supplementary material for: Heterogeneity in transmission parameters of hookworm infection within the baseline data from the TUMIKIA study in Kenya
Source: Parasit Vectors. 2019 Sep 16;12:442. doi: 10.1186/s13071-019-3686-2 (PMC6745791; doi:10.1186/s13071-019-3686-2)
Supplement: Supplementary file 1 — Additional file 1: Text S1. An analysis of cluster level age profiles. Figure S1. a Total log-likelihood for prevalence gradient, m across all clusters (MLE: m = 0:0012/year). b Histogram of MLE prevalence gradients for the 119 clusters. Individual MLE values are subject to an uncertainty of c.0:0015/year. [file 13071_2019_3686_MOESM1_ESM.pdf]

Additional file 1: Text S1. Cluster age profiles

To check for the existence of a consistent age profile pattern of prevalence across clusters in the baseline data, we employ a simple model of age-dependent prevalence, $p_i(a)$, in cluster i,

$$p_i(a; m) = \bar{p}_i + m(a - \bar{a}_i)$$

where m is the gradient of prevalence with age and $\bar{p}_i$ and $\bar{a}_i$ are cluster-specific intercept values. The value $\bar{p}_i$ is the overall prevalence in cluster i from the data and

$$\bar{a}_i = \frac{1}{n_i} \sum_j a_{ij}$$

where n_i is the sample size from cluster i and a_{ij} is the age of the jth individual in the ith sample. This definition has the advantage that average prevalence from the model across the age points in the data always matches that from the data itself. We calculate a log-likelihood for each cluster,

$$\mathcal{L}_i(m) = \sum_{j \in S_i^+} \ln p_i(a_{ij}; m) + \sum_{j \in S_i^-} \ln(1 - p_i(a_{ij}; m))$$

where S_i^+ and S_i^- are the sets of the ages of all egg-positive and egg-negative individuals in the ith sample, respectively. The total log-likelihood is

$$\mathcal{L}(m) = \sum_i \mathcal{L}_i(m).$$

Fig S1A shows the log-likelihood for the age profile prevalence gradient over all clusters, $\mathcal{L}(m)$. The maximum likelihood estimator (MLE) for the gradient is $m = 0.0012$/year with a very small degree of uncertainty, implying a prevalence difference of approximately 6% across a 50 year age difference. This matches the gradient seen in the age profile of the data over all (Fig 1D in the main text). From the point of view of individual clusters, however, there is considerable variability. Fig S1B shows a histogram of the MLE values for gradients in individual clusters. Values cluster around the overall best fit gradient, but are clearly not significantly different from zero, which would imply no age structure. Additionally, the posterior distributions for cluster-level gradient estimates have standard deviations of around ≈ 0.0015/year. These observations, together with the additional simplicity and fewer parameters, support the decision to use a transmission model without age structure.

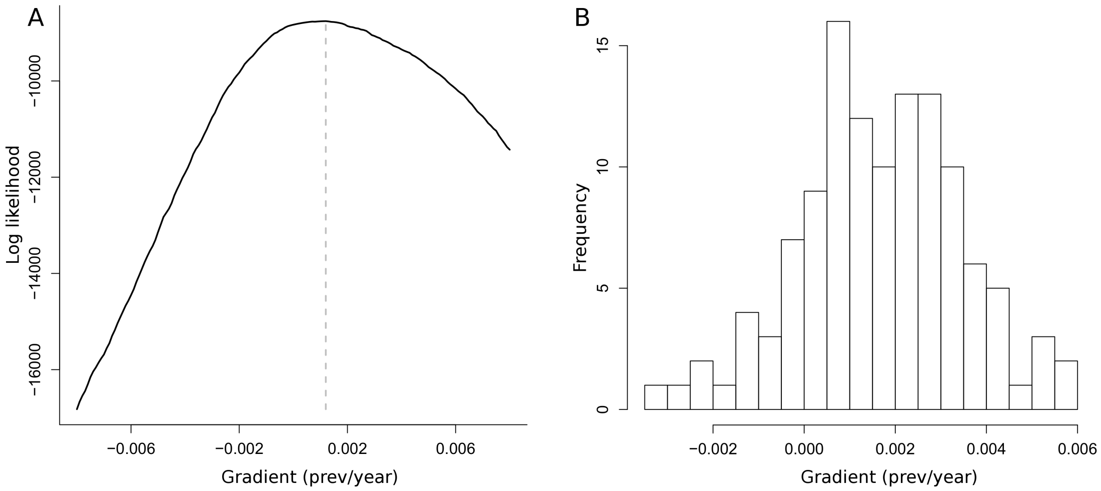

Figure S1: a) Total log-likelihood for prevalence gradient, m across all clusters (MLE: $m = 0.0012$/year). b) histogram of MLE prevalence gradients for the 119 clusters. Individual MLE values are subject to an uncertainty of ≈ 0.0015/year
