# Supplementary material for: Heterogeneity in transmission parameters of hookworm infection within the baseline data from the TUMIKIA study in Kenya
Source: Parasit Vectors. 2019 Sep 16;12:442. doi: 10.1186/s13071-019-3686-2 (PMC6745791; doi:10.1186/s13071-019-3686-2)
Supplement: Supplementary file 2 — Additional file 2: Text S2. Analysis and derivation of likelihood functions that connect the model to data. [file 13071_2019_3686_MOESM2_ESM.pdf]

Additional file 2: Text S2. Prevalence and intensity likelihoods

In order to construct likelihoods for the prevalence and intensity data for a given cluster, it is necessary to calculate (or estimate) the probability distribution for the quantity as predicted by the model.

Calculations are based on the assumption that the worm burden in a host is the result of acquiring fertilised eggs that have an equal probability of being male or female. Heterogeneity in the acquisition rates of worms by hosts results in a negative binomial distribution across the population. As a result, the joint probability of N worms of which n are female is

$$P(n, N) = \mathrm{Bin}(n; N, p)\mathrm{NegBin}(N; 2M, k) \quad \text{(S1)}$$

where M is the mean burden of female worms. $\mathrm{Bim}(\cdot; N, p)$ is binomial distribution of N trials with probability p and $\mathrm{NegBin}(\cdot; 2M, k)$ is a negative binomial distribution with mean $2M$ and aggregation parameter, k. The marginal distribution of female worms is negative binomial with mean M and aggregation k,

$$P(n; M, k) = \mathrm{NegBin}(n; M, k).$$

Equation 1 in the Methods section of the main text describes the evolution of the mean female worm burden, $M(t)$ over time under the effects of parasite transmission and pre-baseline rounds of of LF treatment.

The presence and intensity of infection is determined from the presence of eggs in stool samples, as counted using the Kato-Katz diagnostic method. Female hookworm only produce eggs when fertilized, so it is necessary to know the probability distribution of fertilized female worms among hosts. We assume that a single male is sufficient to fertilize all females in a given host. From the joint probability (equation S1), the probability of n_f fertile females reduces to

$$P(n_f; M, k) = \mathrm{NegBin}(n; M, k) - \mathrm{NegBin}(n_f; 2M, k)\left(\frac{1}{2}\right)^{n_f}, \quad n_f \geq 1, \quad \text{(S2a)}$$

$$P(0; M, k) = 2\left(\frac{k}{M+k}\right)^k - \left(\frac{k}{2M+k}\right)^k. \quad \text{(S2b)}$$

The number of countable eggs generated by fertilized female worms is also a stochastic process, with a negative binomial distribution [1]. The probability of counting E eggs from n_f fertilized females in a host is

$$P(E; n_f, \lambda, k_e, \gamma) = \mathrm{NegBin}(E; \bar{E}, k_e), \quad \bar{E} = \lambda n_f \exp(-\gamma n_f) \quad \text{(S3)}$$

where λ is the net egg count per female and γ parmeterizes the density-dependent fecundity of egg production. The higher the density of female worms in a host the fewer countable eggs each produces. As a result, a zero egg count can arise even when fertilized females are present. The probability of observing no eggs is

$$P_0(M, \Theta) = \sum_{n_f=0}^{\infty} P(0; n_f, \lambda, k_e, \gamma)P(n_f; M, k)$$

where Θ represents a vector of all parameters. As a result, $1 - P_0$ is the observed hookworm prevalence. Hence, for the ith cluster with sample sizeN^i_{Tot} and number of positive individuals, n^i_+, the prevalence likelihood contribution is

$$\mathcal{L}^P_i = \mathrm{Bin}(n_+; N^i_{Tot}, 1 - P_0(M(t_0), \Theta))$$

where $M(t_0)$ is the mean worm burden at the baseline, t_0, generated by the model.

The probability distribution for individual egg count is formed by compounding the distribution for egg count given a known population of fertilized females (equ. S3) and the distribution of fertilized female worms (equ. S2), and hence cannot be conveniently calculated. Consequently, the

resulting form for the distribution used to calculate the intensity likelihood is not in a convenient form. However, it is possible to calculate the mean and variance of the distribution, which can be used to create an approximation to the true distribution.

The expected egg count for an individual is given by

$$\bar{E} = \sum_{n_f=0}^{\infty} \lambda n_f z_f^n P(n_f; M, k)$$

where $z = exp(-\gamma)$. This can be expressed simply in terms of the probability generating function for a negative binomial, G. Let $G(Q; M, k)$ is the probability-generating function for the NB with mean M and aggregation k.

$$G(Q; M, k) = \sum_{n=0}^{\infty} Q^n \mathrm{NegBin}(n; M, k) = \left[\frac{1}{1 + M(1-Q)/k}\right]^k$$

The mean egg count can therefore be written as

$$\bar{E} = \lambda Q \frac{\partial}{\partial Q} G(Q, M, k)\bigg|_{(z,M,k)} - \lambda Q \frac{\partial}{\partial Q} G(Q, M, k)\bigg|_{(z/2,2M,k)}$$

which is

$$\bar{E} = \frac{\lambda M z}{[1 + M(1-z)/k]^{k+1}} - \frac{\lambda M z}{[1 + 2M(1-z/2)/k]^{k+1}}$$

The distribution of individual egg counts is a compound of the distribution of egg counts for a given population of fertilized females and the distribution of fertilized females across the population of hosts. The variance of E can therefore be expressed as

$$\mathrm{Var}[E] = \mathrm{Var}_{n_f}[\mathrm{E}[E(n_f)]] + \mathrm{E}_{n_f}[\mathrm{Var}[E(n_f)]] \tag{S4}$$

For the current distributions, this simplifies to

$$\mathrm{Var}[E] = \left(1 + \frac{1}{k_e}\right) \mathrm{E}_{n_f}[\lambda^2 n_f^2 z^{2n_f}] - \bar{E}^2 + \bar{E}.$$

The expectation term can be evaluated using the negative binomial probability generating function to yield

$$\mathrm{Var}[E] = \left(1 + \frac{1}{k_e}\right)\left(\frac{(k+1)\lambda^2 M^2 z^4}{k[1 + M(1-z^2)/k]^{k+2}} + \frac{\lambda^2 M z^2}{[1 + M(1-z^2)/k]^{k+1}}\right) - \tag{S5}$$

$$\left(1 + \frac{1}{k_e}\right)\left(\frac{(k+1)\lambda^2 M^2 z^4}{k[1 + M(2-z^2)/k]^{k+2}} + \frac{\lambda^2 M z^2}{[1 + M(2-z^2)/k]^{k+1}}\right) - \bar{E}^2 + \bar{E}. \tag{S6}$$

which, while complex, is exact.

Being a compound of two negative binomial distributions, one of which may have a high degree of aggregation, the egg count distribution is likely to be a highly skewed fat-tailed distribution. To achieve a less skewed distribution for likelihood calculation, we fit to the total count in the cluster sample. For the shape of the distribution, we assume a negative binomial, which hopefully captures the expected skewness of the true distribution. Hence, for cluster i with total egg count E_{Tot} across a sample size of n_i, the model distribution will have mean $n_i \bar{E}(\Theta)$ and variance $n_i \mathrm{Var}[E](\Theta)$. The likelihood of the intensity data is given by

$$\mathcal{L}_i^I = \mathrm{NegBin}(E_{Tot}; \bar{E}_i(M, \Theta), k_i(\Theta))$$

where

$$k_i = \frac{n_i \bar{E}}{\mathrm{Var}[E] - \bar{E}}.$$

The total log-likelihood is given by the sum of the prevalence and intensity likelihoods across all clusters,

$$\mathcal{LL} = \sum_i \ln \mathcal{L}_i^P + \ln \mathcal{L}_i^I$$

References

[1] de Vlas, S. J., Gryseels, B., van Oortmarssen, G. J., Polderman, A. M. and Habbema, J. D. F. (1992). A model for variations in single and repeated egg counts in schistosoma mansoni infections. Parasitology. 104, p451.
