# Supplementary material for: Heterogeneity in transmission parameters of hookworm infection within the baseline data from the TUMIKIA study in Kenya
Source: Parasit Vectors. 2019 Sep 16;12:442. doi: 10.1186/s13071-019-3686-2 (PMC6745791; doi:10.1186/s13071-019-3686-2)
Supplement: Supplementary file 3 — Additional file 3: Text S3. Details of the likelihood distribution and the quality of fit of the model. Table S1. MLE and ranges for global parameters from truncated likelihood distribution. Figure S2. Marginal distribution of the likelihood sample. Figure S3. Joint parameter distribution for two individual clusters. Figure S4. Model mean prevalence and total egg count for each cluster. Figure S5. Distribution of model mean prevalences and total egg counts arising from likelihood sample for two individual clusters. Figure S6. Mean and 90% credible intervals for R0 and k for each cluster as sampled from the likelihood distribution. Figure S7. Comparison of model fit to data with MLE and mean parameters. Figure S8. Pairs plot of mean R0 and other parameters with and without likelihood cut-off. Figure S9. Mean and 90% credible intervals for parameter estimates with and without likelihood truncation. Figure S10. Correlation between R0 and k within the truncated likelihood sample. Figure S11. Fit to data for mean and MLE parameters from the truncated likelihood. [file 13071_2019_3686_MOESM3_ESM.pdf]

Additional file 3: Text S3. Likelihood statistics

The likelihood parameter distribution is difficult to interpret as it is a function of R_0 values for each cluster and 4 global parameters. To get an idea of the shape of the distribution, we need reduce the number of dimensions. One way to do this is to display the mean of R_0 across all clusters (Fig S2). The key features of the distribution are clear. The maximum likelihood estimator (MLE) is highly skewed with respect to all the parameters. As a result, the MLE will typically lie outside any credible interval constructed from the likelihood distribution. The skewness of the likelihood is a consequence of the existence of a transmission breakpoint and the requirement that the model have a endemic equilibrium at baseline. As a result, small changes in parameter values close to the breakpoint in the 'wrong direction' can cause the endemic solution to vanish, giving rise to a asymmetry in the parameter distribution. Another feature of the distribution is the concentration of log-likelihood values in the sample that are far from the maximum likelihood (ML) value. The probability distribution of log-likelihood values is approximately χ^2 with degrees of freedom equal to the number of parameters involved. In the current case, the high dimensionality of the likelihood leads to a distribution in which the majority of the likelihood values explored are far below the MLE by approximately 100.

It is also instructive to look at individual clusters. Each cluster has a only 3 varying parameters; R_0, λ and k. Figure S3 shows a sample from the likelihood distributions from two randomly chosen clusters, 15 and 50. The maximum likelihood estimator (MLE) parameter values are extremes within these samples, lying at the highest k and λ values and the lowest values of R_0. As a result, the MLE values for R_0, λ and k fall outside the 95% credible interval generated from the likelihood distribution. The likelihood support for these values is weak for any particular cluster, but much stronger when all are considered together. Asymmetry of the likelihood distribution is primarily due to the need for a stable endemic parasite population. The existence of the break-point in transmission dynamics means that endemic parasite populations do not exist below a critical value of R_0, leading to a lower 'cut-off' in the R_0 distribution in the likelihood which is further reflected in the other parameters.

The overall quality of the fit represented by the likelihood distribution can be gauged by evaluating the mean model prevalence and mean model total egg count for each cluster for the parameter sets in the likelihood MCMC sample. Fig S4 shows the MLE and 95% credible interval for mean prevalence and total egg count alongside the data points for each cluster. For the majority of clusters, the correspondence between data and MLE model output is good and the MLE value is found within the 95% confidence intervals. Fig S5 shows the details of one good and one poor correspondence between the model and data (clusters 15 and 50, respectively). For cluster 15, the MLE model outputs lie near the mean of the likelihood distribution values even though the parameters themselves are outliers within the likelihood. For cluster 50, however, it is clear that the model struggles to match the prevalence and particularly the intensity data simultaneously.

Figure S6 gives an alternative marginal view of the likelihood sample, showing

average values and 90% credible intervals of R_0 and k by cluster. As suggested by Figure S3, mean parameter values differ markedly from the MLE values, with mean R_0 much higher than MLE values in all clusters and both k and λ values being lower. Credible intervals for R_0 values are extremely wide in contrast to those for the aggregation parameters k. Using the mean parameter values from the likelihood distribution instead of the MLE values gives the fit seen in Fig S7A (in contrast to the MLE fit in panel B). Total log-likelihood for the mean parameter values is -1208, a drop of about 90 with respect ot the maximum likelihood and a clear drop in fit quality. More significantly, the generally much higher R_0 values result in much heavier worm burdens, with the maximum mean worm burden in a cluster around 350 worms per host in comparison to around 40 for MLE parameters. This is clearly not biologically plausible and is at odds with measured worm burdens unless the sensitivity of expulsion techniques for ascertaining worm burdens have exceptionally low sensitivities [1].

The high R_0 values and large worm burdens are associated with lower log-likelihood values. These can be removed by truncating the likelihood distribution below a threshold likelihood value. Fig. S8A shows the pair-wise correlations between log-likelihood, the global parameters and the mean reproduction number across all clusters (to reduce the dimensionality of the distribution) for the full distribution (A) and a distribution truncated at -1190 (B). Within this part of the distribution, there is clearly a strong, linear positive correlation between k_L, k_U and λ and a negative correlation between those parameters and mean R_0. Fig. S9 shows the mean and ranges of R_0 and k for individual clusters. Mean R_0 is strongly correlated to egg count, with values ranging from 1.5 to 3.5. The range of R_0 is mostly confined below about 5. The correlation between R_0 and k across the truncated likelihood are shown in Fig S10. We have normalised the R_0 distribution for each k value on the x-axis to attempt to compensate for the clumped nature of the fitted aggregation values. The distribution shows a distinct correlation between the two parameters, mean R_0 increasing approximately linearly with increasing k (decreasing aggregation). Mean parameter values from the truncated likelihood distribution give a fit to data shown in Fig S11. Log-likelihood for the mean parameter fit is 37 below the maximum likelihood with a comparable quality of fit over all clusters. In this case, the maximum mean female worm burden among clusters is 35, within the ranges found in literature [1]. For the lowest likelihood in the truncated distribution, maximum mean female worm burden is around 40.

Based on this approach, we can define parameter ranges for optimal fit combined with realistic mean worm burdens (See table 1). For R_0, mean values for clusters are roughly correlated with mean egg count, running linearly from about 1.5 to 3 as mean cluster egg count goes from approximately zero to 30 (see Fig S9). Ranges for individual cluster R_0 values can be judged from the Fig S9. The variability within these ranges is strongly constrained by the correlations visible in Fig. S8B.

Parameter	MLE	Range
k_L	0.048	[0.03,0.048]
k_U	0.278	[0.14,0.278]
λ	3.06	[2.2,3.06]

Table 1: MLE and ranges for global parameters from truncated likelihood distribution.

References

[1] Turner, H. C., Truscott, J. E., Bettis, A. A., Shuford, K. V., Dunn, J. C., Hollingsworth, T. D., et al. (2015). An economic evaluation of expanding hookworm control strategies to target the whole community. Parasit Vectors. 8:570.

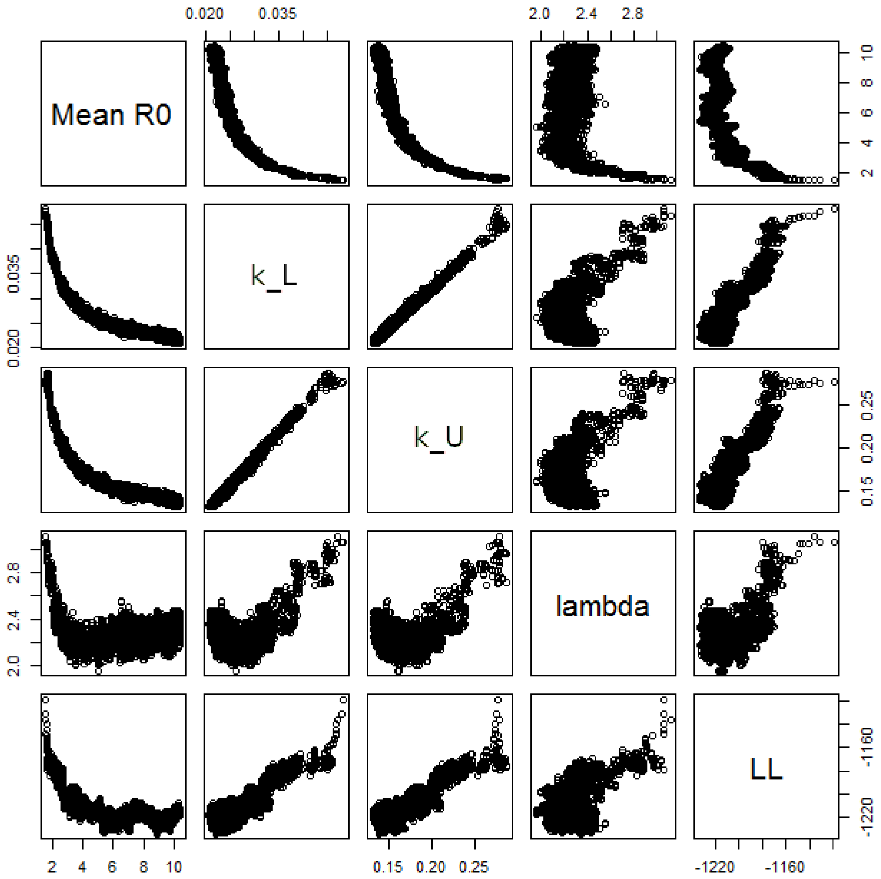

Figure S2: Marginal distribution of the likelihood sample.

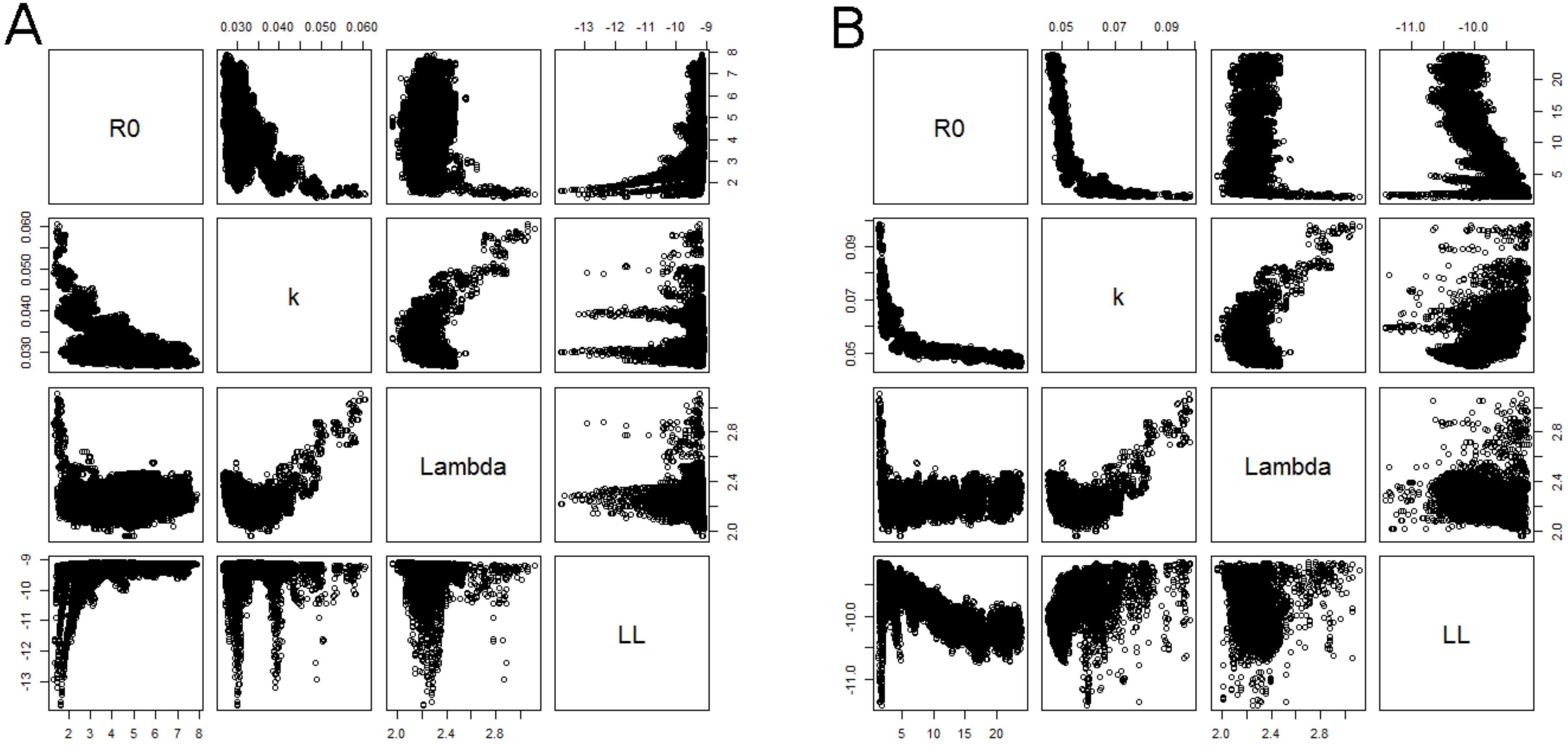

Figure S3: Joint parameter distribution for single clusters 15 (A) and 50 (B). The maximum likelihood parameter estimate is at the largest values of both k and λ and the lowest value of R_0. For individual clusters, the support for these values is not strong.

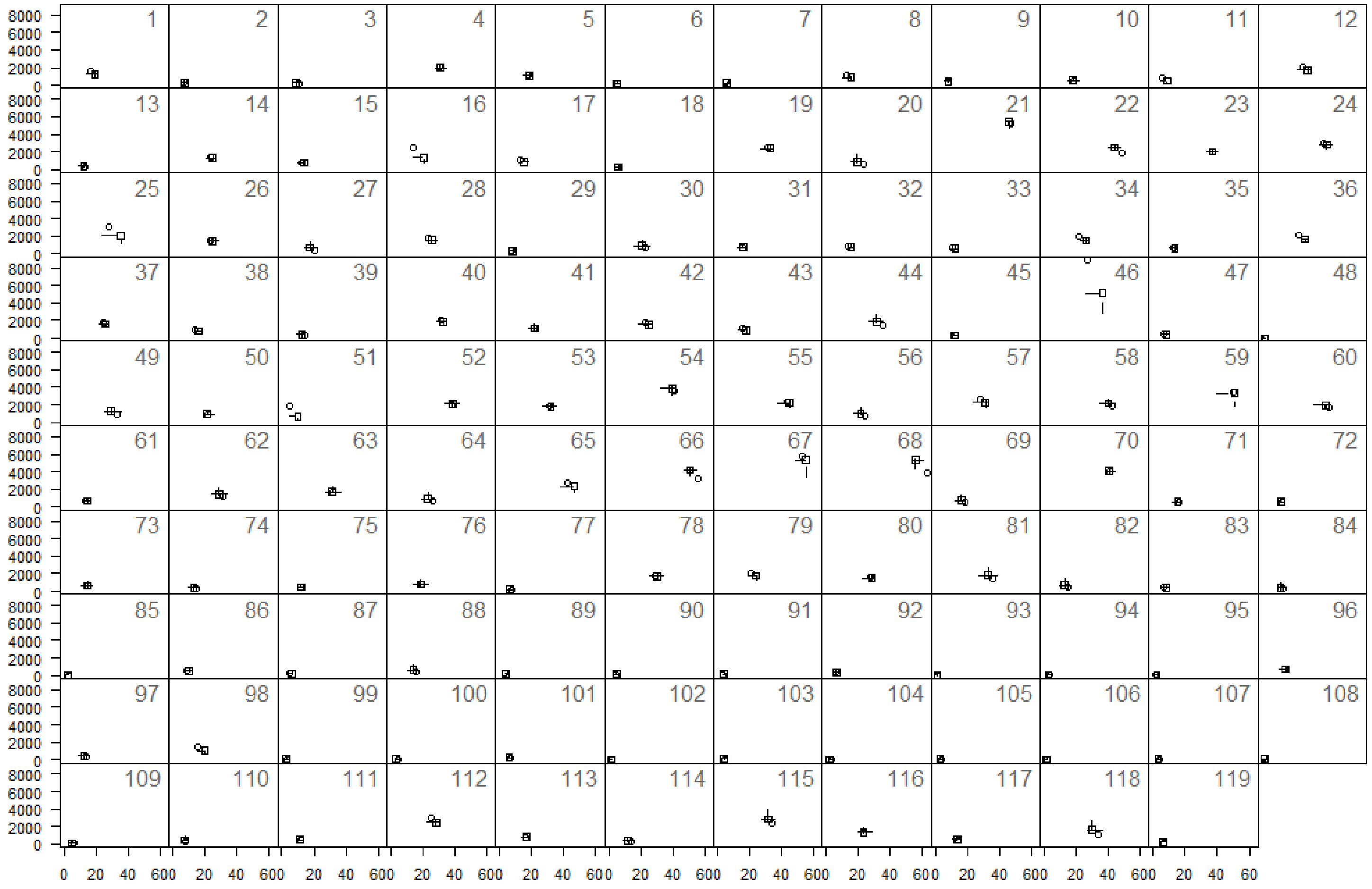

Figure S4: Model mean prevalence and total egg count for each cluster for parameter sets from the likelihood distribution. MLE shown as squares and 95% credible intervals shown as line segments. Equivalent data points shown as circles.

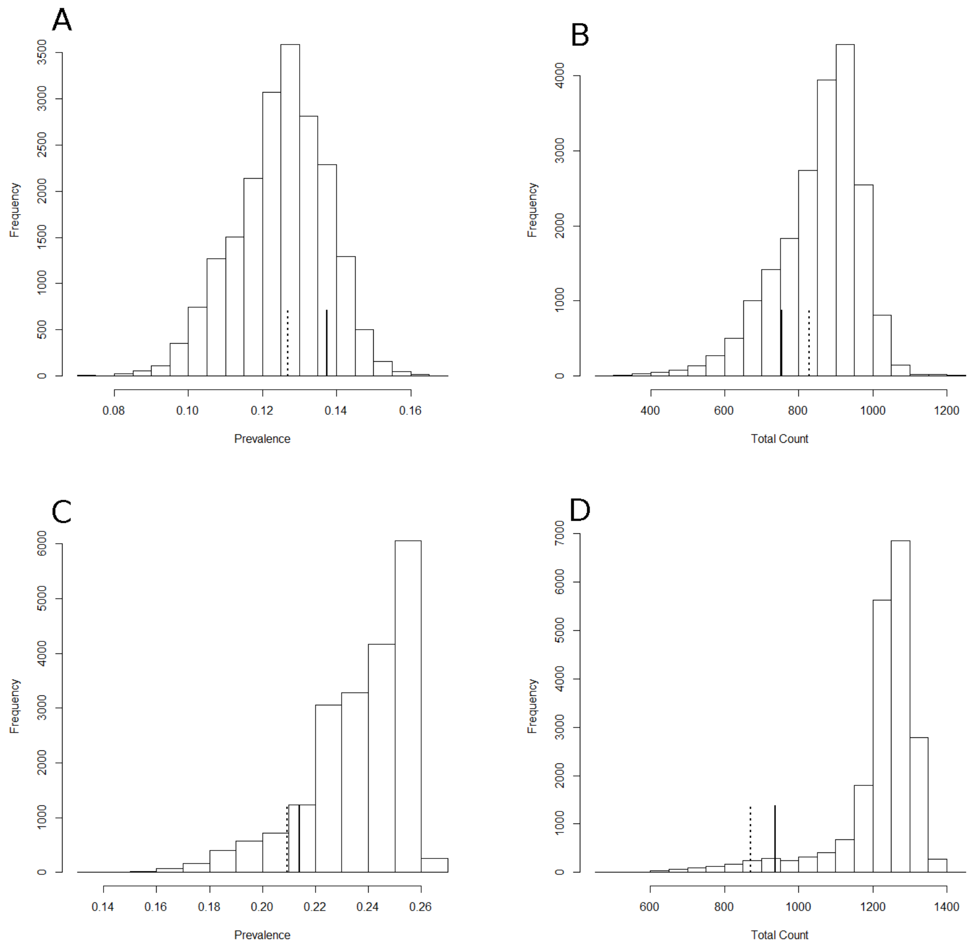

Figure S5: Distribution of model mean prevalences and total egg counts arising from likelihood sample for clusters 15 (A and B) and 50 (C and D). Panels A and C show mean prevalence distribution and panels B and D show mean total count distribution. Dotted line and solid line indicate MLE value and data value, respectively.

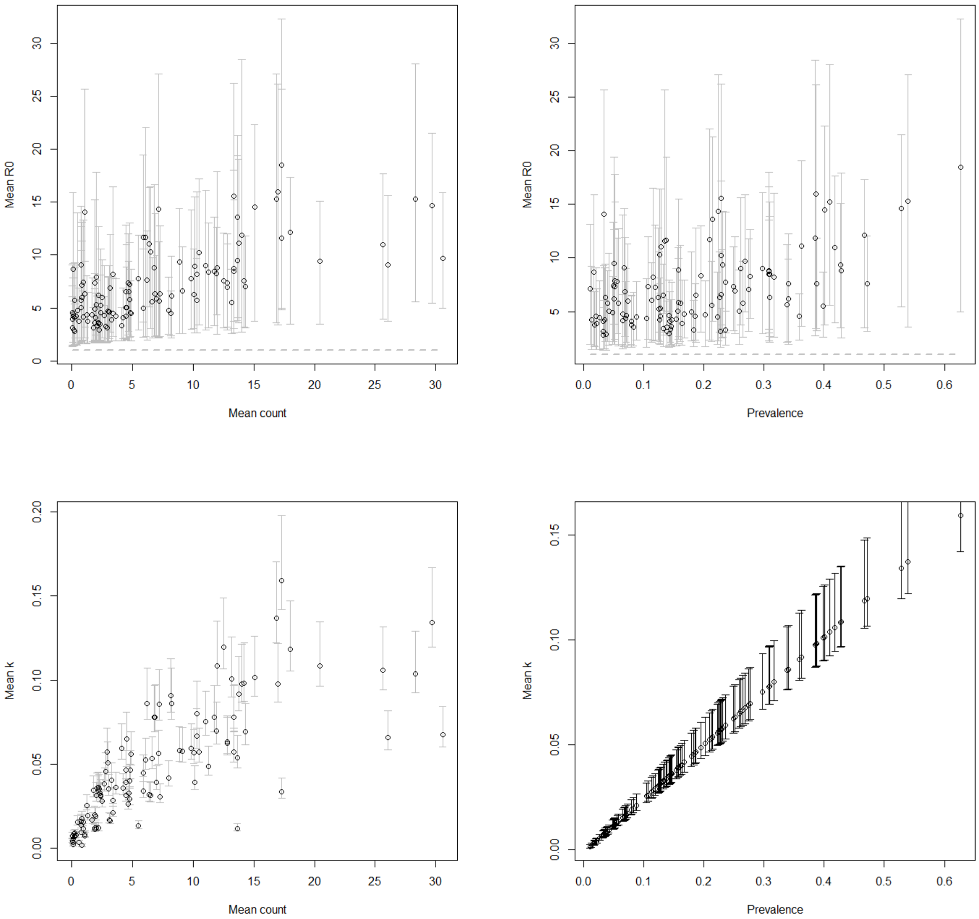

Figure S6: Mean and 90% credible intervals for R_0 and k for each cluster as sampled from the likelihood distribution.

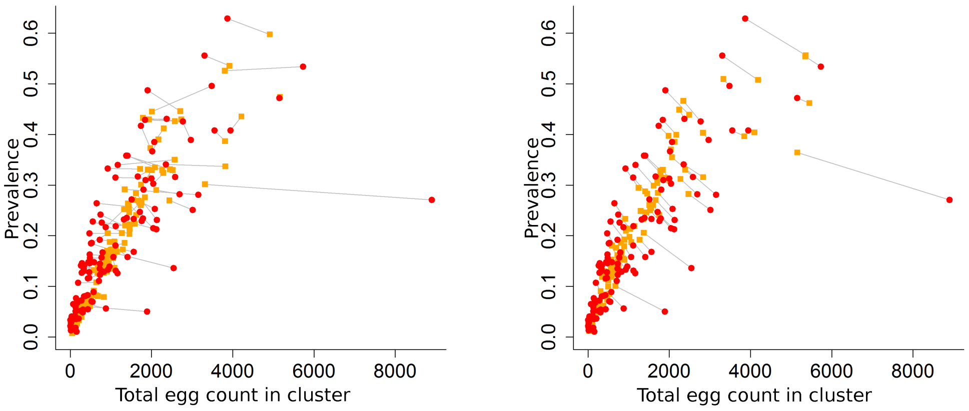

Figure S7: Fit generated by A) mean parameters from the likelihood sample and B) the MLE parameter set. Red circles represent data points and yellow squares mean model fits.

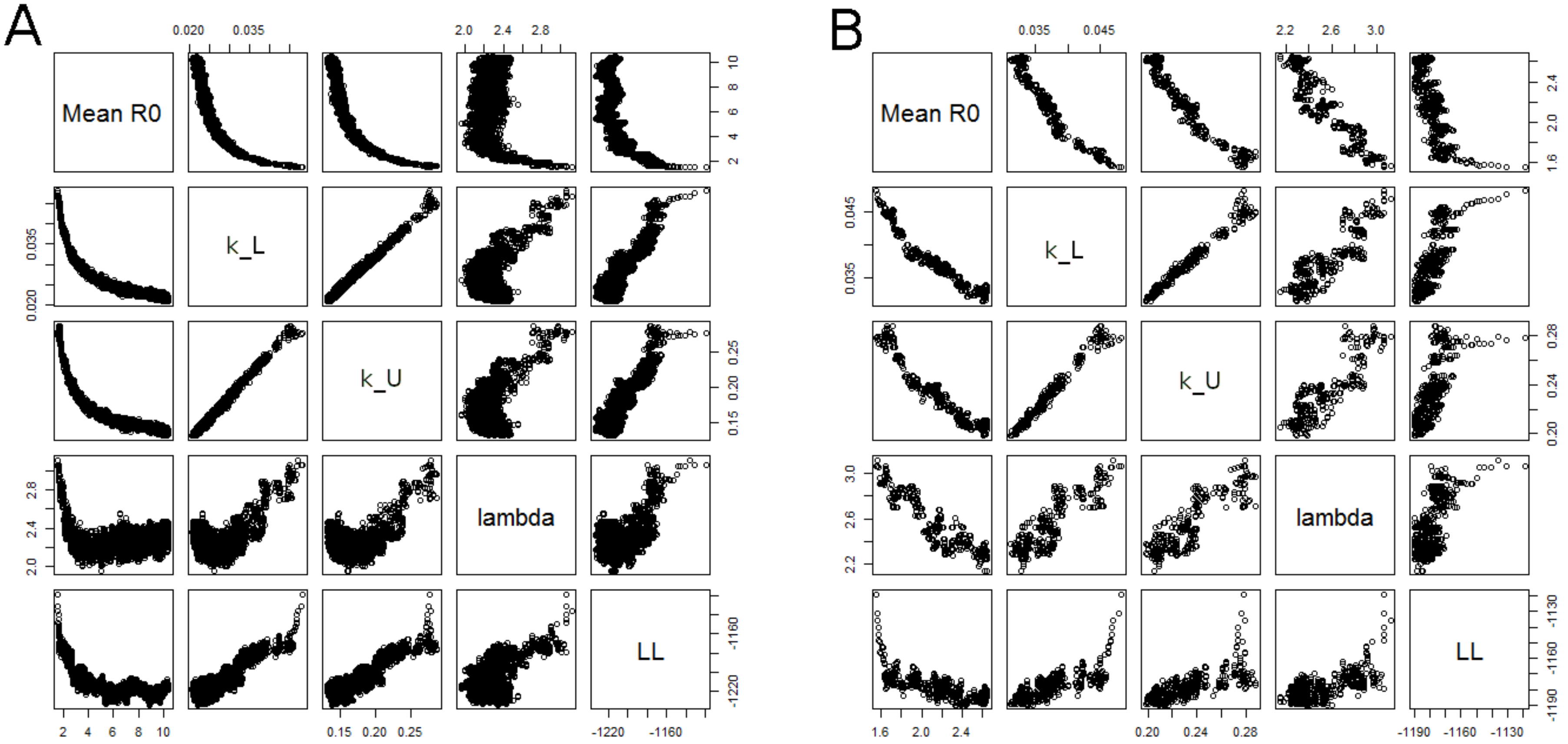

Figure S8: Pairs plot of mean R0 and other parameters with and without likelihood cut-off.

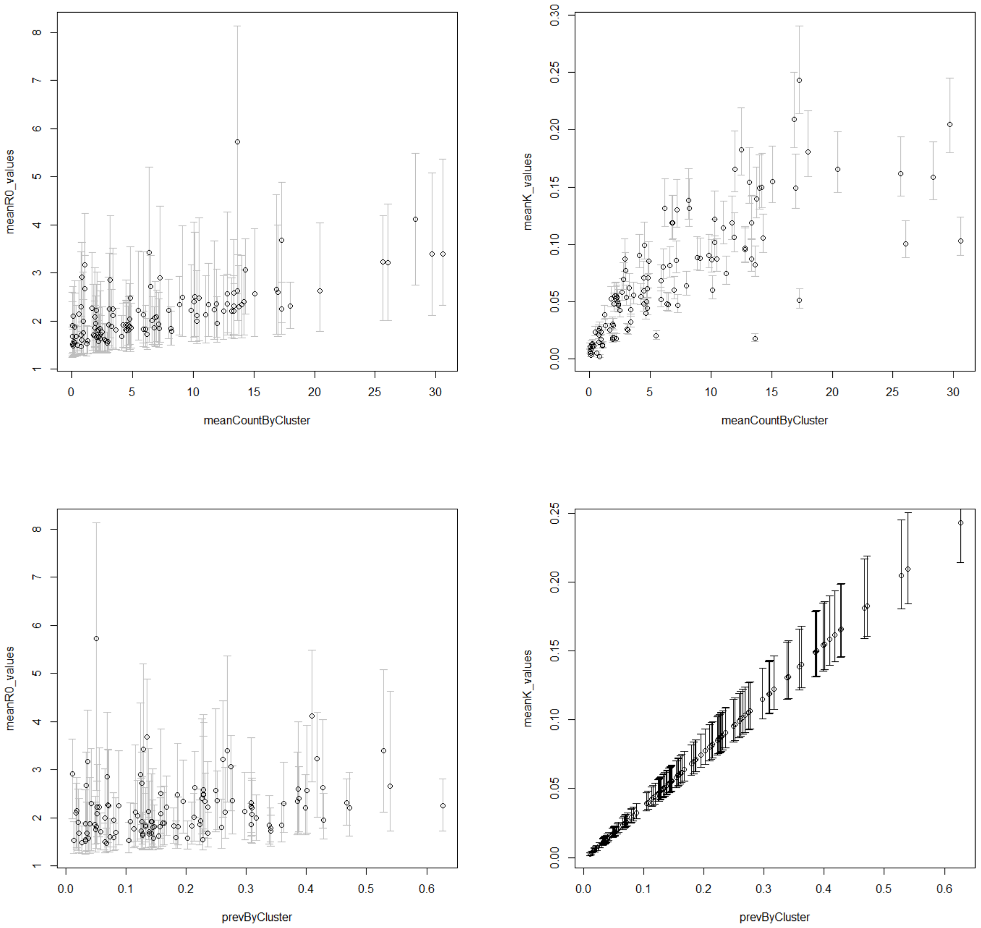

Figure S9: Mean and 90% credible intervals for R_0 and k for each cluster as sampled from the likelihood distribution, truncated at -1190.

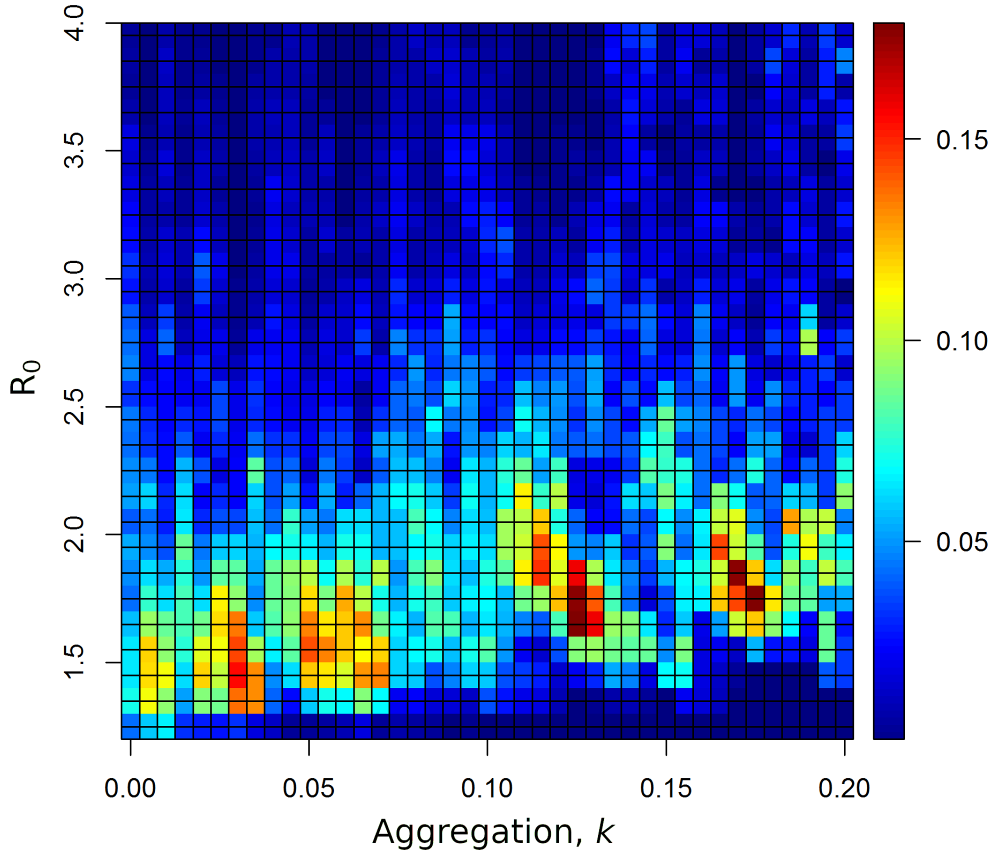

Figure S10: Correlation between R_0 and k within the truncated likelihood sample. For each k value on the x-axis, the associated distribution of R_0 values is normalised.

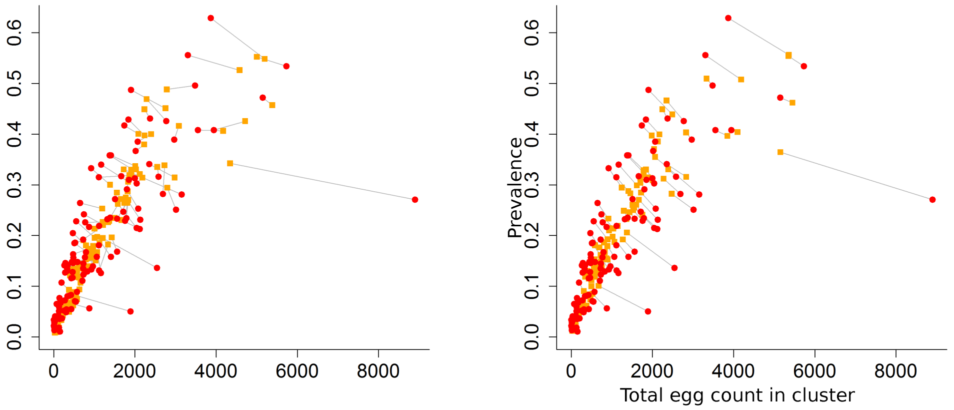

Figure S11: Fit generated by A) mean parameters from the likelihood sample truncated at log-likelihood of -1190 and B) the MLE parameter set. Red circles represent data points and yellow squares mean model fits.
