# Supplementary material for: Heterogeneity in transmission parameters of hookworm infection within the baseline data from the TUMIKIA study in Kenya
Source: Parasit Vectors. 2019 Sep 16;12:442. doi: 10.1186/s13071-019-3686-2 (PMC6745791; doi:10.1186/s13071-019-3686-2)
Supplement: Supplementary file 4 — Additional file 4: Text S4. Full description of covariates for GLM model fitting. Table S2. Landcover (16 variables). [file 13071_2019_3686_MOESM4_ESM.docx]

**Additional file 4: Text S4.** **Climate-Socioeconomic Variables - a full description of covariates for GLM model fitting**

**Socioeconomic (2 variables) wealth score and % sanitation access**: Baseline data regarding household wealth and access to sanitation was collected as part of the Tumikia baseline household questionnaire. For this analysis the mean of the household specific PCA wealth score (categorical) was taken to obtain a cluster level wealth score. To create a cluster level sanitation score, the proportion of households with ‘access to sanitation’ (binary) was used.

**Open access data used, included the variables:**

- **Temperature** (mean annual temperature)
- **Rainfall** (mean annual rainfall)
- **Elevation**
- **Population density**
- **Landcover**

To obtain a cluster level estimate for mean annual temperature, precipitation, elevation, and population density across each cluster, an average score was taken for all data points within the cluster boundaries. This does result in a decrease in accuracy and variability across the cluster, however this is also the case for our prevalence/intensity/R0 estimates, as in most cases multiple villages have been combined to form a single cluster.

**Additional file 4: Table S2. Landcover (16 variables)**

| Code | Definition | % Coverage in Tumikia |
| --- | --- | --- |
| X10 | Cropland, rainfed | 26.83 |
| X30 | Mosaic cropland (>50%) / natural vegetation (tree, shrub, herbaceous cover) (<50%) | 39.68 |
| X50 | Tree cover, broadleaved, evergreen, closed to open (>15%) | 10.55 |
| X90 | Tree cover, mixed leaf type (broadleaved and needle leaved) | 18.58 |
| X40 | Mosaic natural vegetation (tree, shrub, herbaceous cover) (>50%) / cropland (<50%) | 1.33 |
| X120 | Shrubland | 2.51 |
| X110 | Mosaic herbaceous cover (>50%) / tree and shrub (<50%) | 0.15 |
| X11 | Herbaceous cover | 14.84 |
| X170 | Tree cover, flooded, saline water | 0.77 |
| X190 | Urban areas | 0.82 |
| X100 | Mosaic tree and shrub (>50%) / herbaceous cover (<50%) | 0.13 |
| X160 | Tree cover, flooded, fresh or brackish water | 3.67 |
| X210 | Water bodies | 0.04 |
| X60 | Tree cover, broadleaved, deciduous, closed to open (>15%) | 0.09 |
| X20 | Cropland, irrigated or post‐flooding | 0.03 |
| X130 | Grassland | 0.002 |

The pixilation of data obtained differed by dataset with the lowest resolution for the temperature and rainfall indicators (30 second data). In cases where the clusters were smaller than the pixels the resolution was increased 10 times – this does not add in finer resolution data but rather increases the number of identical pixels within the origin 1 pixel. For each numeric indicator the mean was taken across all pixels that fell into a cluster boundary. In the case of landcover, the proportion of each classification of landcover was taken for a cluster.
